# Characterization of 3D heterocellular spheroids of pancreatic ductal adenocarcinoma for the study of cell interactions in the tumor immune microenvironment

**DOI:** 10.3389/fonc.2023.1156769

**Published:** 2023-07-14

**Authors:** Giulio Giustarini, Germaine Teng, Andrea Pavesi, Giulia Adriani

**Affiliations:** ^1^ Singapore Immunology Network (SIgN), Agency for Science, Technology and Research (ASTAR), Singapore, Singapore; ^2^ Institute of Molecular and Cell Biology (IMCB), Agency for Science, Technology and Research (ASTAR), Singapore, Singapore; ^3^ Mechanobiology Institute, National University of Singapore, Singapore, Singapore; ^4^ Department of Biomedical Engineering, National University of Singapore, Singapore, Singapore

**Keywords:** pancreatic ductal adenocarcinoma, cancer, spheroids, *in vitro* model, heterocellular, tumor immune microenvironment, macrophages, cytokines

## Abstract

Pancreatic ductal adenocarcinoma (PDAC) is one of the deadliest malignancies nowadays. The available chemo- and immunotherapies are often ineffective in treating PDAC due to its immunosuppressive and highly desmoplastic tumor immune microenvironment (TIME), which is hardly reproduced in the existing preclinical models. The PDAC TIME results from a peculiar spatial organization between different cell types. For this reason, developing new human models recapitulating the tissue organization and cell heterogeneity of PDAC is highly desirable. We developed human 3D heterocellular tumor spheroids of PDAC formed by cancer cells, endothelial cells, pancreatic stellate cells (PSC), and monocytes. As a control, we formed spheroids using immortalized epithelial pancreatic ductal cells (non-cancerous spheroids) with cellular heterogeneity similar to the tumor spheroids. Normal spheroids containing endothelial cells formed a complex 3D endothelial network significantly compromised in tumor spheroids. Monocyte/macrophages within the 4-culture tumor spheroids were characterized by a higher expression of CD163, CD206, PD-L1, and CD40 than those in the non-cancerous spheroids suggesting their differentiation towards an immunosuppressive phenotype. The heterocellular tumor spheroids presented a hypoxic core populated with PSC and monocytes/macrophages. The 4-culture tumor spheroids were characterized by spatial proximity of PSC and monocytes to the endothelial cells and a cytokine signature with increased concentrations of CXCL10, CCL2, and IL-6, which have been observed in PDAC patients and associated with poor survival. Further, 4-culture tumor spheroids decreased the concentrations of T-cell chemoattracting cytokines, i.e., CCL4, CCL5, and CXCL9, when compared with the non-cancerous spheroids, revealing a critical immunosuppressive feature of the different types of cells forming the tumor spheroids. Our results showed that the 4-culture tumor spheroids better resembled some critical features of patients’ PDAC TIME than monoculture tumor spheroids. Using the proposed human 3D spheroid model for therapy testing at the preclinical stage may reveal pitfalls of chemo- and immuno-therapies to help the development of better anti-tumor therapies.

## Introduction

Pancreatic Ductal Adeno-Carcinoma (PDAC) represents 2.6% of the incidence of cancer worldwide, nonetheless, 4.7% of the mortality (1). Available therapies, namely chemotherapies, immunotherapies, and radiotherapies for treating PDAC are characterized by a poor response associated with minimal survival (2, 3). The lack of effective therapies undoubtfully contributes to the 10.8% 5-year relative survival of PDAC patients (4). Within the tumor immune microenvironment (TIME), cells, extracellular matrix, and soluble factors play a pivotal role in the resistance to therapies (5). The available preclinical models for PDAC, including both *in vivo* and *in vitro* models, suffer limitations in identifying clinically effective therapies due to their inability to fully recapitulate the complex and heterogeneous TIME observed in patients. The development of 3D heterocellular PDAC spheroids represents a step forward in predicting chemoresistance, better simulating key features of PDAC pathophysiology compared to 2D *in vitro* tests (6). Pancreatic stellate cells (PSC) are the main source of extracellular matrix (ECM) in PDAC (7) and have been shown to play a key role in tumor vascularization and in establishing an immunosuppressed environment in PDAC (8, 9). PSC have similar functions to the hepatic stellate cells and their transcriptional profile in response to inflammation is unique among the other fibroblastic populations in pancreatic cancer (10), dramatically expanding during carcinogenesis in an orthotopic murine model (11). Recently, Helms et al. demonstrated that PSC-derived myofibroblasts, rather than other fibroblasts of different origins, have a non-redundant capability to shape the desmoplastic PDAC TIME (10), although representing only 10 to 15% of the cancer-associated fibroblasts in an orthotopic murine model (8, 9). In two different models, PSC showed their capability to increase proliferation and expression of vimentin (mesenchymal marker) whereas decreasing the expression of E-cadherin (epithelial marker) in the pancreatic cancer cell line PANC-1 when co-cultured in a 3D spheroid (12, 13). Adding MRC-5 immortalized fibroblastic cells of fetal lung origin to 3D PDAC spheroid models have shown to implement certain features observed in patients’ TIME, such as the differentiation of monocyte to M2-like macrophages (14). However, is still unclear whether PSC influences cancer cells and primary human monocytes/macrophages in 3D co-culture systems. This uncertainty arises because human PSC-derived fibroblasts exhibit protective effects on pancreatic cancer cells, which are not observed in co-cultures with MRC-5 fibroblastic cells, (15).

Macrophages of both fetal and hematopoietic stem cell ontogeny have been observed within the PDAC TIME. Embryonically- and hematopoietic stem cell-derived macrophages exert different functions, such as shaping the fibrotic processes and regulating the immune responses, respectively, as demonstrated in an orthotopic murine model of PDAC (16). In line with these findings, clinical trials have shown that the dual inhibition of chemotactic receptors (CCR2 and CCR5) for the recruitment of monocytes has a beneficial effect on the anti-tumor immunity and chemotherapeutic response in PDAC compared to either strategy alone. This evidence tailored a fundamental immunosuppressive and drug-resistance role for monocytes within the PDAC TIME, underlining their involvement in response to therapies (Tomás-Bort et al., 2020).

An abundant cell type within the PDAC TIME is represented by endothelial cells, even though tumor vascularization is often heavily compromised (17). To include endothelial cells (EC), a previous study has combined human umbilical vein EC (HUVEC) with lung fibroblasts and different pancreatic cancer cell lines to form 3D PDAC spheroids. The heterogeneous spheroids containing the three cell types showed stronger chemoresistance of cancer cells to standard therapies (i.e., doxorubicin and gemcitabine) when compared with spheroids formed by only cancer cells, underlining important synergisms between endothelial cells and other cells in the spheroids to reproduce drug resistance as observed in patients (18).

However, none of the 3D heterocellular PDAC spheroids mentioned above or similar models (6, 15, 19) included immune cells within the PDAC heterocellular spheroids.

We hypothesized that *in vitro* co-culture of monocytes/macrophages with cancer, endothelial, and stellate cells in 3D spheroids could reproduce a microenvironment similar to what was observed in the immune niche of different tissues (20, 21), in which cells establish a partnership promoting mutual changes in transcription and survival, shaping their differentiation and activation within the spheroids.

Therefore, here we present the first 3D heterocellular human spheroid model of PDAC formed using PANC-1 cancer cells, pancreatic stellate cells (PSC), endothelial cells (EC) and peripheral blood mononuclear cell (PBMC)-derived monocytes. Combining immunofluorescence, flow cytometry, and cytokine analysis, we characterize the viability, proliferation, cytokine concentrations, and monocyte differentiation within the spheroids. We observed that the 4-culture spheroids were characterized by spatial proximity of PSC, monocytes, and EC, which formed a 3D endothelial network and were characterized by a cytokine signature resembling the one observed in PDAC patients with poor survival. The model included control spheroids formed using h-TERT-human pancreatic epithelial nestin-expressing cells (HPNE), which served and will serve to validate differences with cancer spheroids. Ultimately, we envision that the cellular interactions within the heterocellular PDAC spheroids containing the four cell types (cancer, stellate, endothelial cells, and monocytes) will better simulate key features of PDAC and our heterocellular spheroids will represent a better tool for more complex microfluidic models studying T cell interaction with the patient’s TIME.

## Material and methods

### Cell culture

PANC-1 (American Type Culture Collection, ATCC), h-TERT-HPNE (human pancreatic epithelial nestin-expressing cells immortalized by transducing with a h-TERT cDNA) (ATCC, Manassas, VA, USA) (22) were maintained in Iscove Modified Dulbecco Media (IMDM) supplemented with 10% fetal bovine serum (FBS, ThermoScientific Cat#10082147) and penicillin/streptomycin (100 U/mL, Invitrogen/Gibco Cat#15140122). Human Pancreatic Stellate Cells (HPaSteC, here referred as PSC) (Gene Etichs Cat#3830, Lot#14358) were maintained in Stellate Cell Medium (SteCM, ScienCell Research Laboratories, Cat#5301) supplemented with 2% FBS (ScienCell Research Laboratories, Cat#0010), 1% stellate cell growth supplement (ScienCell Research Laboratories, SteCGS, Cat#5352) and 1% antibiotic solution (ScienCell Research Laboratories, Cat#0503). Human umbilical vein endothelial cells (HUVEC, Lonza, C2519AS, Lot#633426) and RFP-HUVEC (Angioproteomie cAP-001RFP, Lot#2021122802) were cultured in EGM-2™ SingleQuot™ containing 0.5 ng/ml VEGF, 5 ng/ml EGF, 10 ng/ml bFGF, 20 ng/ml long R3-IGF-1, 22.5 μg/ml heparin, 1 μg/ml ascorbic acid, 0.2 μg/ml hydrocortisone, gentamicin (1/1000 dilution) and 2% FBS. All cells were cultured in 75 and 175 cm^2^ tissue culture treated flasks in a humidified atmosphere composed of 95% air and 5% CO_2_ and a temperature of 37°C. Cells were passaged every 72 h using 0.25% (PANC-1 and HPNE) or 0.05% (HUVEC and PSC) Trypsin-EDTA (Gibco, Thermo Fisher Scientific, Cat# 25300054).

### Generation of GFP-PANC-1 cells

The generation of GFP-tagged PANC-1 cells was achieved using lipofectamine 2000-mediated transfection following manufacturer’s instruction. Briefly, PANC-1 cells were cultured and maintained in appropriate growth media before transfection with an enhanced green fluorescent protein (eGFP) encoding plasmid (pEGFP-C1 EGFP-3XNLS, cat. N. #58468, Addgene) encapsulated in Lipofectamine 2000. The lipofectamine-GFP plasmid complex was added to the PANC-1 cells and allowed to incubate for 48 h. Following the transfection, the GFP-tagged PANC-1 cells were monitored under a fluorescence microscope to confirm successful GFP expression and subsequently FACS sorted to enrich the positive population.

### Human blood cells

Ethical approval for obtaining healthy human volunteer blood cones was obtained by the institutional ethical review board under the Project No.: 201306-04 and all subjects provided written informed consent.

### Monocyte isolation

Peripheral blood mononuclear cells (PBMC) were isolated from blood cones using a density gradient of Ficoll/Paque PLUS (GE Healthcare, Marlborough, MA, USA). The content of the blood cones was diluted 40x in phosphate buffer saline (PBS) and placed onto a layer of Ficoll/Pacque (density 1.077 g/L) before centrifuging at 900 x g, at room temperature (RT) for 20 min. The obtained PBMC layer was collected using a sterile pipette and washed with Ca^-^Mg^-^ PBS before incubation with red blood cell lysis (155 mM NH_4_Cl, 10 mM KHCO_3_, 0.1 mM EDTA) for 5 min at RT. Cells were washed in Ca^-^Mg^-^ PBS and prepared for cryopreservation using Bambanker™ (Fujifilm Wako Chemicals U.S.A. Corporation, Richmond, VA, USA). On the day of the experiment, cryopreserved PBMC suspension was used for the isolation of untouched monocytes using Pan Monocyte Isolation kit (Miltenyi Biotec, Bergisch Gladbach, Germany). The isolated cells were characterized by the expression of CD14 and CD16 in a CD45^+^/CD3^-^ gate. Monocytes represented more than 95% of the total CD45^+^ cells.

### Cell labeling

Cells were collected in tubes and counted before washing them once in PBS. Cells were spun down at 300 x g for 5 min and the supernatant discarded. PBS containing CellTrace™ Violet (cat. n. C34557, Thermo Fisher, dilution 1/1000), CellTracker™ Deep Red (cat. n. C34565, Thermo Fisher, dilution 1/1000) and CellTracker™ Green CMFDA (cat. n. C2925, Thermo Fisher, dilution 1/500) was used to stain the different cell types according to the requirements of the experiment. The cell pellet was resuspended in PBS containing the dyes and incubated at 37°C. After 30 min, 5 ml of medium containing FBS was used to stop the staining reaction. Other 5 ml of medium containing FBS was used to wash the cells before resuspending them in EGM-2 for spheroid formation.

### Hanging-drop spheroid formation

Heterogeneous cell suspensions for the spheroid formation were obtained by mixing either PANC-1 or HPNE with PSC, HUVEC and PBMC-derived monocytes following the ratios shown in **Table 1**. The seeding number of PANC-1 cells was determined to achieve a minimum radius of approximately 250 µm and a viability above 90% of the spheroids at day 7. EGM-2 was adopted as medium for the spheroid formation after we assessed no significant changes in the cell number of PANC-1, HPNE, HPaSteC and monocytes in 2D culture using EGM-2 compared to the respective recommended media (**Supplementary Figure 1**). The PANC-1 cell number was kept constant to better assess the contribution of each cell type on cancer cells. The cell ratio to PANC-1 cell was tuned to observe the arrangement of endothelial cells into a three-dimensional (3D) network. With this aim, several preliminary ratios were investigated for EC, PSC and monocytes. We selected the ratios supporting the formation of the 3D EC network. The seeding number of HPNE cells was determined by their ability to form spheroids with a radius similar to the PANC-1 spheroids, while ensuring their viability remains above 90%. The other cell types in the HPNE spheroids were kept in the same number as for the PANC-1 spheroids. Cells were resuspended in EGM-2 and seeded using a custom-made polydimethylsiloxane (PDMS) support for the formation of spheroids with the hanging drop technique. Spheroids were formed in 4 or 7 days depending on the experiment.

**Table 1:** Composition of PANC-1 spheroids (ratios).

Condition	PANC-1		HPaSteC		HUVEC		Monocytes	
	Ratio	Cell number	Ratio	Cell number	Ratio	Cell number	Ratio	Cell number
**PANC-1**	1	(1500)	–	(-)	–	(-)	–	(-)
**PANC-1+PSC**	1	(1500)	2	(3000)	–	(-)	–	(-)
**PANC-1+EC**	1	(1500)	–	(-)	2	(3000)	–	(-)
**PANC-1+Mono**	1	(1500)	–	(-)	–	(-)	4	(6000)
**PANC-1+PSC+EC**	1	(1500)	2	(3000)	2	(3000)	–	(-)
**PANC-1+PSC+Mono**	1	(1500)	2	(3000)	–	(-)	4	(6000)
**PANC-1+EC+Mono**	1	(1500)	–	(-)	2	(3000)	4	(6000)
**PANC-1+PSC+EC+Mono**	1	(1500)	2	(3000)	2	(3000)	4	(6000)

-, Cell type not included in the spheroid.

### Immunofluorescence

At day 4 or 7, spheroids were spun down at 300 x g for 1 min and each drop was assessed for the formation of the spheroid by an inverted microscope. All the formed spheroids were collected in 1.5 mL microcentrifuge tubes and spun down for 1 min at 300 x g. Supernatant was removed, and spheroids were incubated with new medium containing a dilution of the membrane impermeable DNA-staining DRAQ7 (Thermo-Fisher Scientific, Waltham, MA, USA) for 30 min at 37°C. Spheroids were washed in PBS before adding PBS containing 4% paraformaldehyde. After 20 min at RT spheroids were washed 3 times with PBS + 1% bovine serum albumin (BSA) before proceeding with the staining. For intracellular staining, spheroids were permeabilized using PBS Ca^-^Mg^-^ + 0.5% Triton-X-100. After 30 min, spheroids were washed two times with PBS Ca^-^Mg^-^ + 1% BSA before incubating them with PBS Ca^-^Mg^-^ + 0.5% Triton-X-100 0.5% + 5% BSA for 3 h. After washing with PBS Ca^-^Mg^-^ + 1% BSA, spheroids were incubated overnight with PBS Ca^-^Mg^-^ + 1% BSA containing one of the following antibodies: rat AlexaFluor594-conjugated anti-human Ki67 antibody (1:100, cat. n. 11-5698-82, EBioscience, Thermo-Fisher Scientific), recombinant anti-human eFluor650-conjugated HIF-1α antibody (1:50, cat. n. 190569, Abcam, Cambridge, UK) and rabbit anti-human purified collagen I antibody (1:50, cat. n. 34710, Abcam, Cambridge, UK). For collagen I immunofluorescent staining, overnight incubation at 4°C with goat anti-rabbit AlexaFluor546 anti-IgG secondary antibody (Fisher Scientific, Carlsbad, CA, USA) in PBS Ca^-^Mg^-^ + 1% BSA. Spheroids were washed 3 times with PBS Ca^-^Mg^-^ + 1% BSA. The spheroids stained for Ki67 were incubated with Hoechst 33342 (1 μg/ml) in PBS + 1% BSA. After 1 h, spheroids were washed with PBS Ca^-^Mg^-^ + 1% BSA before proceeding with image acquisition using an inverted confocal microscope Olympus FV1000 (Olympus, Tokyo, Japan).

### Flow cytometry

Spheroids were collected in 1.5 mL Eppendorf tubes and washed with PBS Ca^-^Mg^-^ + 1% BSA before proceeding with cell dissociation. Dissociation was performed in 2 steps: 1) incubation with RPMI containing 1 mg/ml Collagenase type IV (Worthington Biochemical Corporation, USA) for 20 min at 37°C; 2) addition of 0.25% trypsin + EDTA (0.53 mM) and further incubation for 20 min. Enzymatic activity was stopped by addition of RPMI containing 10% FBS. Cells were washed with fluorescence‐activated cell sorting buffer (PBS containing 0.5% BSA, 0.05% NaN_3_, 0.5 mM EDTA) and prepared for flow cytometry staining. Cells were first stained with LIVE/DEAD^®^ Fixable Dead Cell Stain (Molecular Probes, Invitrogen, Carlsbad, CA, USA) followed by incubation with fragment crystallizable region receptor (FcR)-blocking antibody to block the FcR. The following antibodies were used for the extracellular staining of cells obtained from the spheroids: mouse PerCP-cy5.5-conjugated anti-human CD86 (clone: 2331) (cat. n. 561129, BD Bioscience, Franklin Lakes, NJ, USA), mouse PE-conjugate anti-human CD68 (clone: Y1/82A) (cat. n. 333808, BioLegend, San Diego, CA, USA), mouse Pacific Orange-conjugated anti-human CD14 (clone: TuK4) (cat. n. MHCD1430, Invitrogen), mouse anti-human eFluor-450 CD206 (clone: 19.2) (cat. n. 48-2069-41, eBioscience), mouse BUV395-conjugated CD45 anti-human (clone: HI30) (cat. n. 563791, BD Bioscience), mouse BUV737-conjugated anti-human CD40 (clone: 5C3)(cat. n. 741847, BD Bioscience), mouse PE-CF594-conjugated anti-human CD163 (clone: GHI/61) (cat. n. 562670, BD Bioscience), mouse PE-cy7-conjugated anti-human CD274 (PD-L1, clone: MH3) (cat. n. 329718, BioLegend), mouse BV605-conjugated anti-human HLA-DR (clone: L243) (cat. n. 307640, BioLegend). For those analysis requiring the intracellular staining, after the cells were stained for the extracellular staining, they were fixed using intracellular fixation buffer (eBioscience). Following procedures reported in the manufacturer’s instructions, the permeabilization buffer was used to incubate the antibodies recognizing α-SMA and HIF-1α. The antibodies used to recognize these proteins are the following: mouse AlexaFluor 488-conjugated anti-human α-SMA (cat. n. 53-9760-82, Invitrogen), recombinant eFluor650-conjugated anti-human HIF-1α (cat. n. 190569, Abcam). After the intracellular staining cells were resuspended in FACS buffer and prepared for acquisition at BD FACS Symphony A3. Data were analyzed using FlowJo analysis software (Tree Star, Inc., Ashland, OR, USA).

### Microscopy

Images of the spheroids were acquired using the inverted confocal microscope Olympus FV1000. Images were processed using IMARIS software (v. 9.7.1, Bitplane). We identified Ki67 positive nuclei using the functions “spots”. PANC-1 nuclei were identified selecting mean intensity of Hoechst 33342 and GFP using background subtraction and considering the fluorescent signal elongation due to the acquisition. Ki67 foci positive cells were considered positive using the intensity sum of the signal emitted by the AlexaFluor594-conjugated anti-human Ki67 antibody within the identified nuclei. Dead cells were excluded based on their mean intensity for DRAQ7.

Analogously, for HIF quantification nuclei were identified using Hoechst 33342 signal using the function “spots” and HIF-AlexaFluor647 signal was identified using a cobalt chloride treated spheroid (positive control). HIF-1α positive control was obtained exposing the formed spheroid to 200 µM cobalt chloride in EGM-2 for 24 h.

Cell composition of the spheroids was identified using the fluorescence of different CellTrackers/CellTracers, GFP-PANC-1 and RFP-HUVEC. Cells were identified using both “spots” and “surface” functions selecting a diameter of cells and a threshold for the mean/median fluorescence intensities after automatic background subtraction and considering the fluorescent signal elongation due to the acquisition. Object to object distance was used to plot distance between different cell types using “Vantage”. Normal spatial distributions of the identified spots were provided by the software. ImageJ software was used to determine the spatial distribution of the different cell types within the HPNE/PANC-1 spheroid. Mean fluorescence of the cells was assessed using the function “plot profile” selecting a region of interest containing the spheroids. Diameter of the spheroids was used to identify an outer and inner region as depicted in **Supplementary Figure 2**. Area under the curve of mean fluorescence was calculated for each region (inner, outer) and the inner/outer ratio of the areas (distribution ratio) plotted as arbitrary unit (A.U.).

### Human cytokine multiplex bead-based assay

The analysis of multiple cytokines in the spheroid formation medium at day 7 was performed using the Luminex technology. For each condition at day 7 after seeding, the spheroid formation medium of 18 spheroids was pooled in a single Eppendorf tube. Spheroids were centrifuged 1 min at 300 x g and supernatant transferred in a new Eppendorf tube. First, the supernatant was centrifuged 5 min at 400 x g. The supernatant was collected and further centrifuged for 10 min at 2000 x g. Supernatant was transferred in a 96 well plate and stored at -80°C until analysis. Supernatant was thawed the day of the assay and prepared for the use of Bio-Plex Pro Human Cytokine Screening Panel 48-Plex (Biorad). Samples were incubated with fluorescent-coded magnetic beads pre-coated with respective antibodies in a black 96 well clear-bottom plate overnight at 4°C. After incubation, plates were washed 5 times with wash buffer (PBS with 1% BSA (Capricorn Scientific) and 0.05% Tween-20 (Promega)). Sample-antibody-bead complexes were incubated with Biotinylated detection antibodies for 1 h and washed 5 times with wash buffer. Subsequently, Streptavidin-PE was added and incubated for another 30 min. Plates were washed 5 times again, before sample-antibody-bead complexes were re-suspended in sheath fluid for acquisition on the FLEXMAP^®^ 3D (Luminex) using xPONENT^®^ 4.0 (Luminex) software. Data analysis was done on Bio-Plex ManagerTM 6.1.1 (Bio-Rad). Standard curves were generated with a 5-PL (5-parameter logistic) algorithm, reporting values for both mean florescence intensity (MFI) and concentration data.

### Statistics

Data are presented as means ± standard error of the mean (SEM) if not differently stated in the figure caption. Statistical significance for comparisons was determined by one- or two-way ANOVA with different *post-hoc* tests. Proper *post-hoc* test is indicated in the figure legend. A p-value less than 0.05 was considered statistically significant. All data are analyzed using GraphPad Prism (version 6.07) software (San Diego, CA, USA).

## Results

### HPNE and PANC-1 cells form viable heterocellular spheroids

**Figure 1:**
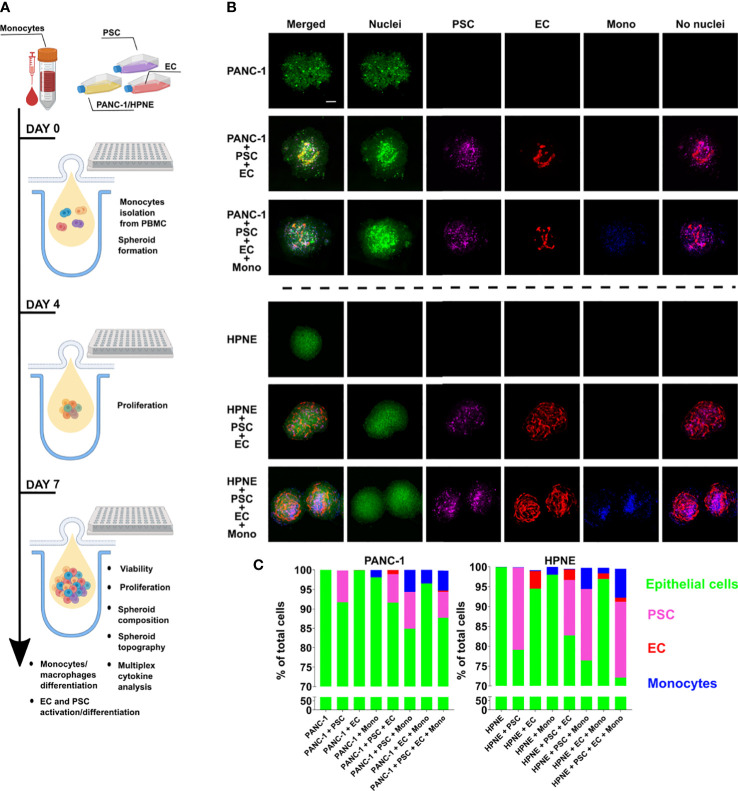
Characterization of size, viability and composition of HPNE and PANC-1 spheroids with different cellular heterogeneity. PANC-1 and HPNE cells were combined with PSC, EC and monocytes following the ratio shown in **Table 1**. **(A)** Schematic illustration of the workflow of spheroid formation and assays performed. **(B)** Representative images of monoculture, 3-culture and 4-culture of PANC-1 and HPNE spheroids at day 7 using confocal imaging (green: nuclei, purple: PSC, red: EC, blue: monocytes); scale bar: 100 μm. **(C)** Parts of whole graph showing the spheroid composition as % of each cell type on the total number of live cells, determined by flow cytometry analysis at day 7.

Heterogenous cell suspensions with the composition indicated in **Table 1** were cultured using a custom-made PDMS layer for the spheroid formation by hanging drop technique (**Figure 1A**). On day 7, the spheroids were collected and prepared for imaging to assess size and viability (**Figure 1B**; **Supplementary Figures 4, 5**). PANC-1 spheroids with lower cell heterogeneity, namely the monoculture PANC-1 spheroids and the bi-culture (2-culture) PANC-1+PSC spheroids, showed a significantly smaller size when compared with the other spheroids containing more cell types with mean radii ranging between 248 μm for monoculture and 318 μm for quadri-culture (4-culture) (**Supplementary Figure 3A**). Among the different conditions, spheroid containing PANC-1+EC showed the highest mean radius of 380 μm. DRAQ7+ cells, representing the non-viable cells with a compromised cell membrane, did not exceed 10% of the total number of cells (**Supplementary Figure 3B**) for all the spheroid compositions, suggesting good viability of the spheroids at the time point assessed. Spheroids obtained using h-TERT HPNE only slightly increased their size accordingly with their heterogeneity without a statistically significant difference among different spheroid compositions (**Figure 1C**). The radii of the HPNE spheroids were shorter than the PANC-1 spheroids with the same heterogeneity.

### Different cell types co-exist and interact in the heterocellular HPNE and PANC-1 spheroids

The cell composition analysis of the spheroids was performed using flow cytometry after spheroid dissociation on day 7 (**Figure 1C**). The cell composition was determined as percentage of each cell type over the total cell number in the spheroid. For PANC-1 spheroids, EC (in red in **Figure 1C**) were detected only in spheroids containing PSC and were averagely of 0.5 and 1% in PANC-1+PSC+EC+Mono and PANC-1+PSC+EC conditions respectively (**Figure 1C**). By contrast for HPNE, EC were detected in all the spheroids (**Figure 1C**). For PANC-1 spheroids, PSC (in purple in **Figure 1C**) were present in all the spheroids. The percentage of PSC in the spheroids was ranging from 6.7 to 9% without significant differences among the spheroid conditions (**Figure 1C**). Similarly, for HPNE spheroids, PSC were ranging between 14 to 20% without significant differences among conditions (**Figure 1C**). For PANC-1 spheroids, monocytes (in blue in **Figure 1C**) at day 7 were increasing their percentages accordingly with the increase in heterogeneity of the PANC-1 spheroids. In particular, we observed that monocytes were ~1% when cultured alone with PANC-1 but their percentage increased to 3% in PANC-1+EC+Mono, and 5% in PANC-1+PSC+Mono and PANC-1+PSC+EC+Mono spheroids (**Figure 1C**). Although a similar percentage increase of monocytes was observed within HPNE spheroids with a higher heterogeneity, monocyte percentage in HPNE+EC+Mono spheroids was not statistically different from the percentage detected in HPNE+Mono spheroids (**Figure 1C**).

### PSC and monocytes distribute in proximity of a disrupted endothelial cell network within the PANC-1 tumor spheroids

Spatial cell organization and cellular functions are interconnected parameters as demonstrated by recent transcriptional and proteomic studies (23). The spatial organization of individual cells into aggregates has been shown to be dependent on self-assembly occurring *via* ligand-receptor interactions (24).

**Figure 2:**
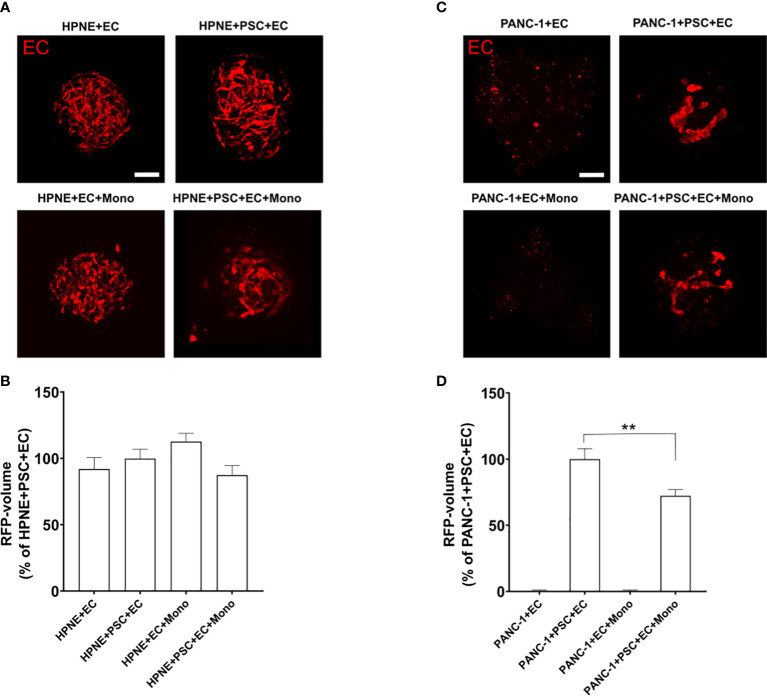
PANC-1 spheroids show a disrupted 3D endothelial network compared to HPNE spheroids. Spheroids were formed using RFP-EC in order to assess by confocal microscopy the EC organization within the spheroids with different cellular composition. **(A)** Representative confocal images of the different PANC-1 spheroids containing RFP-EC at day 7. **(B)** Bar plot of the volume of the EC network quantified as percentage of the RFP volume of PANC-1+PSC+EC spheroids. Data shows mean ± SEM. Statistical significance for comparisons was determined by one-way ANOVA with Dunnett’s *post-hoc* test **p<0.01 when compared to the volume of PANC-1+PSC+EC. **(C)** Representative confocal images of the HPNE spheroids containing RFP-EC at day 7. **(D)** Bar plot of the volume of the EC network quantified as percentage of the RFP volume of HPNE+PSC+EC spheroids. Data shows mean ± SEM. Statistical significance for comparisons was determined by one-way ANOVA with Dunnett’s *post-hoc* test.

It has been demonstrated that vasculature in PDAC TIME has compromised functions due to the highly desmoplastic TIME. The molecules contained in the ECM take part in the physical stress causing the vessels within the tumor to collapse (25). The structures of the 3D endothelial network did not exhibit a lumen and, for this reason, they were not defined as vessels. However, we assessed the formation of endothelial networks within our spheroids and whether the presence of other cell types influences the EC organization using RFP-labeled EC (**Figure 2**). The RFP-EC were imaged within the spheroids by confocal microscopy (**Figures 2A, C**) and the complexity of the endothelial cell network was quantified by measuring the volume of the RFP fluorescence signal as percentage of the RFP-EC volume in HPNE+PSC+EC (**Figure 2B**) or PANC-1+PSC+EC (**Figure 2D**) for HPNE and PANC1 spheroids, respectively. We observed that HPNE spheroids collected at day 7 showed a complex 3D endothelial network with similar volumes in all the spheroids (**Figures 2A, B**). By contrast, PANC-1 spheroids collected at day 7 had no endothelial network formation when cultured without PSC (**Figures 2C, D**). The representative images of the RFP fluorescence signal showed a distinct reduction of the 3D endothelial network complexity in PANC-1 spheroids when compared to HPNE spheroids (**Figures 2A, C**). Indeed, a disrupted endothelial network was observed at day 7 in PANC-1 spheroids containing PSC (**Figure 2C**) compared to the HPNE spheroids with the same cell composition (**Figure 2A**). In addition, PANC-1+PSC+EC+Mono spheroids showed a significant decrease of ~28% in the RFP fluorescence volume compared to the PANC-1+PSC+EC spheroids not containing monocytes (**Figures 2C, D**).

By utilizing the 3D endothelial cell network as a reference, we evaluated the spatial cellular organization of PANC-1 4-culture spheroids that emerged from cell-cell interactions during the formation of the spheroids.

Distances between different cell types were calculated using the function “object-to-object” statistics and plotted using “Vantage” with IMARIS software. EC were identified as “surfaces” whereas the other cell types were identified as “spots” and the software quantified the distance of each spot from the EC surface.

**Figure 3:**
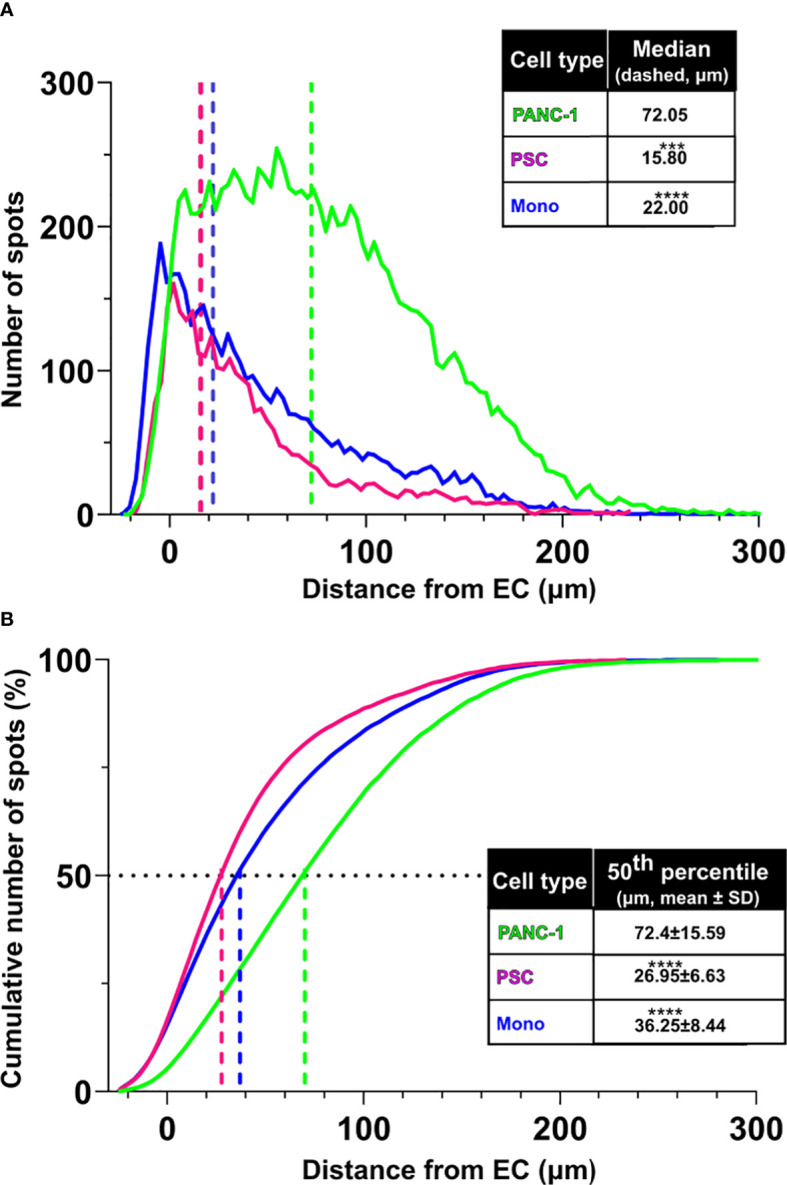
PSC and monocytes distribute in proximity of the EC network. **(A)** Distribution of cell distance from the EC surface by cell type (green: PANC-1, purple: PSC, blue: monocytes. Statistical significance for comparisons was determined by one-way ANOVA with Kruskal-Wallis’ *post-hoc* test. **(B)** Plot of the cumulative distribution of the distance of PANC-1, PSC and monocytes from the EC network. The plot shows also the distance from EC at which 50% of the total number of each cell type is detected. Mean values of the 50^th^ percentile distance was used for statistical significance using one-way ANOVA with Dunnett’s *post-hoc* test. ****p<0.0001, ***p<0.001 when compared to distance from EC of the PANC-1 cells.

The distribution of the distances between the different cell types showed that PSC and monocytes were at a median distance of respectively 15.80 and 22.00 μm from the EC surface, whereas PANC-1 cells showed a significantly greater median distance of 72.05 μm from the EC surface (**Figure 3A**). The cumulative distribution function of PSC showed that 50% of the identified PSC were located within a 26.95 μm mean distance from the EC surface (**Figure 3B**) and this was not significantly affected by the presence of monocytes within the aggregates (data not shown). The cumulative distribution function of monocytes showed that 50% of these cells were located within a 36.25 μm mean distance from the EC surface. By contrast, the distribution of PANC-1 cells revealed that 50% of the cells were located at a significant greater mean distance of 72.40 μm from the EC surface compared to PSC and monocytes (**Figure 3B**).

### PSC and monocytes are located in the core of PANC-1 tumor spheroids

We assessed the spatial cell distribution within the spheroids by dividing the spheroid in two concentric areas: an outer (OUT) and an inner (IN) area. We defined the inner area as the circle having half of the total spheroid radius and having the same center of the spheroid. The remain area of the spheroid was considered the outer area (**Supplementary Figure 2**). In these areas we calculated the area under the curve (AUC) of the fluorescent signal of each cell type. The AUC IN/OUT ratio of the fluorescent signal of each cell type provides an understanding of the preferred location of the cells within the spheroid.

**Figure 4:**
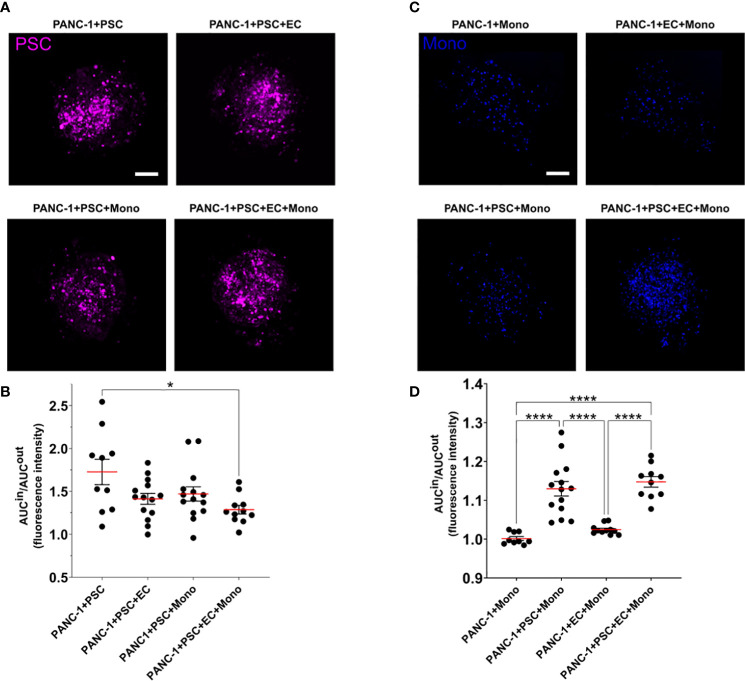
PSC and monocytes spatial distribution within the PANC-1 spheroids. **(A)** Representative images of PANC-1 spheroids at day 7 with different compositions acquired using confocal imaging showing only the fluorescent signal of PSC (purple). Scale bar: 100 µm. **(B)** Dot plot of the ratio between the area under the curve (AUC) of the PSC fluorescent signal in the outside or inside regions of the spheroids. Data are shown as mean ± SEM. Statistical significance for comparisons was determined by one-way ANOVA with Tukey’s *post-hoc* test. *p<0.05 when compared to PANC1+PSC spheroids. **(C)** Representative images of PANC-1 spheroids at day 7 with different compositions acquired using confocal imaging showing only the fluorescent signal of monocytes (blue). Scale bar: 100 µm. **(D)** Dot plot of the ratio between the area under the curve (AUC) of the monocyte fluorescent signal in the outside or inside regions of the spheroids. Data are shown as mean ± SEM. Statistical significance for comparisons was determined by one-way ANOVA with Tukey’s *post-hoc* test. ****p<0.0001.

Independently of the other cells in the PANC-1 spheroids, the integrated fluorescence intensity signal of PSC in the inner region of the spheroids was greater than the one detected in the outer region with an average AUC IN/OUT distribution ratio above 1.2 (**Figures 4A, B**) suggesting that PSC are preferentially located in the inner area for all spheroid conditions. A statistically significant 25% decrease of the AUC IN/OUT ratio was observed in the 4-culture when compared to the PANC-1+PSC spheroids which presented the greatest mean ratio among the tested conditions (**Figure 4B**) suggesting that the PSC tend to distribute more homogeneously within the spheroids when in 4-culture.

Monocyte distribution within the PANC-1 spheroids was characterized by a statistically significant higher AUC IN/OUT distribution ratio when co-cultured with PSC in 3-culture and 4-culture spheroids with an average AUC IN/OUT distribution ratio above 1.1, whereas PANC-1 spheroids not containing PSC had a distribution ratio approximately equal to 1 (**Figures 4C, D**), suggesting that the monocytes were preferentially distributed within the inner area of the spheroids only when in co-culture with the PSC.

### PANC-1 4-culture spheroids produce peripheral collagen I and express HIF-1α in the spheroid’s core

Collagen I represents one of the most abundant molecules in the ECM of PDAC and it is often identified as the molecule responsible of the desmoplastic reaction associated with a reduced survival in patients (26). Hypoxic responses are often observed in collagen-rich conditions in PDAC mostly due to the cross-linked molecules of collagen which exert a compression on the vasculature (27). Hypoxia-related gene transcription is regulated by a class of transcription factors called the hypoxic inducible factors (HIFs), and in particular, the ubiquitous HIF-1α. The activation of the hypoxic responses has a role in shaping the TIME by mediating the transcription of genes involved in cancer cell metabolism and modulating the release of cytokines and growth factors (28). Thus, we characterized the expression of collagen I and HIF-1α in our spheroids with different cellular heterogeneity.

**Figure 5:**
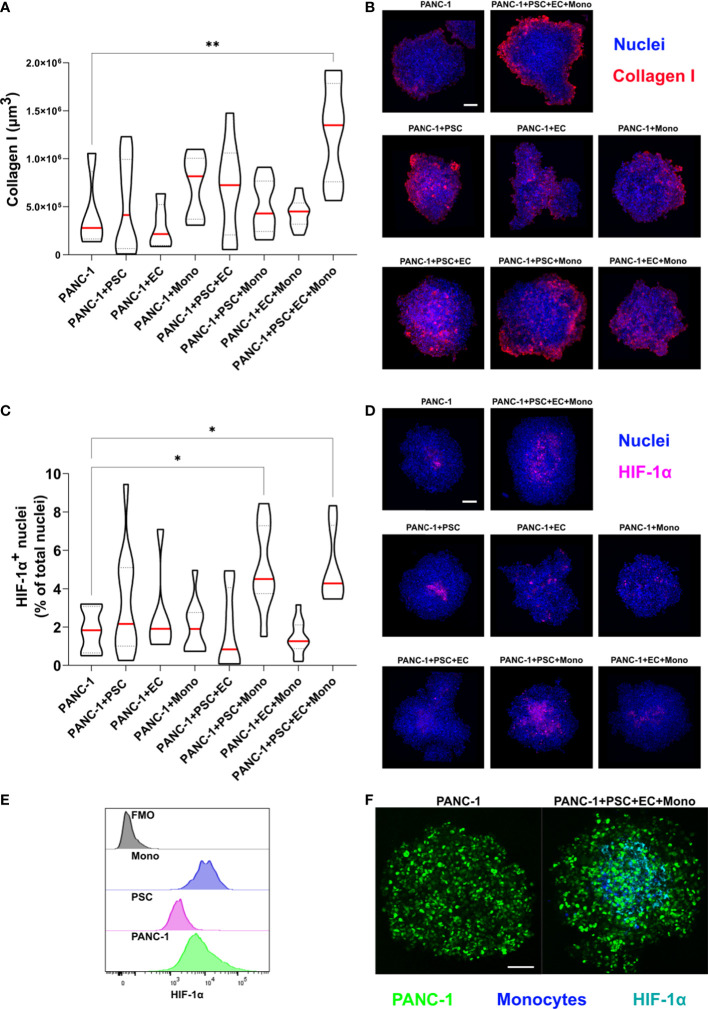
PANC-1 4-culture spheroids are characterized by peripheral collagen I deposition and a hypoxic core. PANC-1 spheroids were collected at day 7 and prepared for immunofluorescent staining with collagen I and HIF-1α antibodies. **(A)** Truncated violin plot showing the volume of collagen I fluorescent signal which was quantified as volume using IMARIS™ (red line: median, dotted line: 25^th^ and 75^th^ quartiles). Statistical significance for comparisons was determined by one-way ANOVA with Dunnett’s *post-hoc* test. **p<0.01 when compared to PANC-1 monoculture spheroids at day 7 **(B)** Representative pictures of PANC-1 spheroids stained with anti-collagen I antibody and Hoechst 33342. (Collagen I: red; blue: nuclei). Scale bar: 100 µm. **(C)** Truncated violin plot showing the number of HIF-1α positive nuclei in the PANC-1 spheroids. Nuclei were identified as spots using IMARIS™ and data were presented as percentage of the total nuclei (red line: median, dotted line: 25^th^ and 75^th^ quartiles). Statistical significance for comparisons was determined by one-way ANOVA with Dunnett’s *post-hoc* test **p<0.01 when compared to PANC-1 monoculture spheroids at day 7. **(D)** Representative pictures of PANC-1 spheroids stained with anti-HIF-1α antibody (nuclei: blue, nuclear HIF-1α: purple). Scale bar: 100 µm. **(E)** Distribution of HIF-1α fluorescent intensity assessed by flowcytometry in the different cell types within PANC-1 4-culture spheroids. **(F)** Representative pictures of monoculture and 4-culture PANC-1 spheroids with similar dimensions stained with HIF-1α (PANC-1: green; HIF-1α: cyan). Scale bar: 100 µm. *p<0.05

The immunofluorescent staining revealed that 4-culture spheroids have a significantly greater expression of collagen I when compared to any other tested PANC-1 spheroid (**Figure 5A**). The collagen I was mainly observed at the periphery of the spheroids, especially in the PANC-1 4-culture spheroids (**Figure 5B**).

In order to quantify the activation of HIF-1α we assessed the percentage of HIF-1α positive nuclei on the total number of nuclei (**Figure 5C**). We observed that PANC-1 monoculture spheroids had a limited percentage of positive nuclei (**Figure 5D**) as well as 2-culture PANC-1 spheroids. By contrast, spheroids containing monocytes had very bright positive nuclei (**Figure 5D**). Interestingly, the combination of PSC and monocytes in the 3- and 4-culture PANC-1 spheroids resulted in a significant increase of the percentage of HIF-1α positive nuclei when compared to the other tested spheroids (**Figure 5C**). Moreover, the HIF-1α positive nuclei of 3- and 4-culture PANC-1 spheroids were mainly located in the core of the spheroids rather than in the outer region (**Figure 5D**).

Using flow-cytometry we investigated the expression of HIF-1α on the cells used to form the PANC-1 4-culture spheroids. As showed in the histogram plot in **Figure 5E**, monocytes had the greater median expression of HIF-1α among the cells within the PANC-1 4-culture spheroids, suggesting the monocytes greatly contributed to the high HIF-1α nuclear expression observed in the PANC-1 3- and 4-culture spheroids.

To assess the size-dependency of the HIF-1α expression, we further formed monoculture spheroids with the same diameter of the 4-culture spheroids by increasing the number of initial PANC-1 cells seeded for spheroid formation. Monocyte embedded in these spheroids were stained with Cell Tracker Violet (CTV). Our results were confirmed because also the bigger PANC-1 monoculture spheroids presented fewer positive nuclei for HIF-1α compared to the PANC-1 4-culture spheroids that presented an evident HIF-1α expression in the core. Among the positive nuclei in the 4-culture we could detect several monocytes although positivity was also detected into PANC-1-GFP cells (**Figure 5F**).

### PSC increase PANC-1 cell proliferation

**Figure 6:**
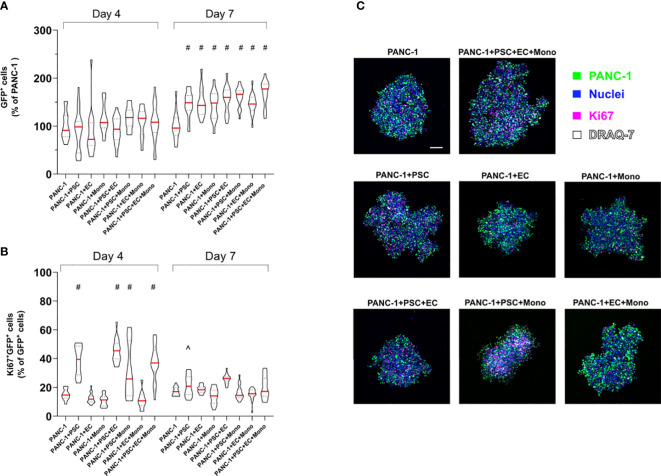
PSC increase PANC-1 proliferation. GFP-PANC-1 cells were used to form the spheroids with different cellular heterogeneity and stained for the detection of Ki67 both at day 4 and 7 and imaged with a confocal microscope **(A)** Truncated violin plot of the live GFP-PANC-1 cells were counted using IMARIS™ software and identified as Hoechst 33342^+^ (blue)/GFP^+^ (green)/DRAQ7^-^ (white) spots. Number of live GFP-PANC-1 cells were plotted as percentage of the number of live GFP-PANC-1 cells in monoculture spheroids. Data are shown as median (red line), 25^th^ and 75^th^ quartiles (dotted lines). Statistical significance for comparisons was determined by two-way ANOVA with Sidak’s *post-hoc* test. ^#^p <0.0001 when compared to PANC-1 monoculture spheroids at day 7. **(B)** Violin plot of the Ki67^+^ cancer cells (purple) plotted as percentage of live GFP-PANC-1 at each condition. Data are shown as median (red line), 25^th^ and 75^th^ quartiles (dotted lines). Statistical significance for comparisons was determined by two-way ANOVA with Sidak’s *post-hoc* test. ^#^p<0.0001 when compared to PANC-1 monoculture spheroids at day 4. ^p<0.01 when compared to PANC-1 monoculture spheroids at day 7. **(C)** Representative confocal images of the heterogeneous spheroids at day 4 labeled with the different markers. Scale bar: 100 µm.

Although we seeded the same number of PANC-1 cells for spheroid formation across the different conditions, we measured an increased number of GFP-PANC-1 cells when they were co-cultured for 7 days in 2-culture, 3-culture and 4-culture with the different combinations of PSC, EC or monocytes (**Figure 6A**). To assess the proliferation of the GFP-PANC-1 cells we measured the Ki67 expression during the spheroid formation at day 4 and day 7. We quantified the proliferating cells by selecting GFP^+^ cells (only PANC-1) expressing nuclear Ki67 and plotted the values as percentage of the total number of GFP-PANC-1 cells at day 4 and day 7 (**Figure 6B**). At day 4, PANC-1 spheroids containing PSC showed an increased percentage of proliferating PANC-1 cells which was averagely double the percentage of proliferating cells observed in PANC-1 only spheroids (**Figures 6B, C**). In contrast, PANC-1 spheroids containing EC or monocyte without PSC did not show any increased Ki67 expression at day 4 (**Figures 6B, C**). At day 7, only PANC-1+PSC showed an increased percentage of Ki67+ positive nuclei when compared to the monoculture spheroids (**Figure 6B**).

### Monocytes increased expression of CD68, CD206, CD163, PD-L1 and CD40 in 4-culture tumor spheroids

**Figure 7:**
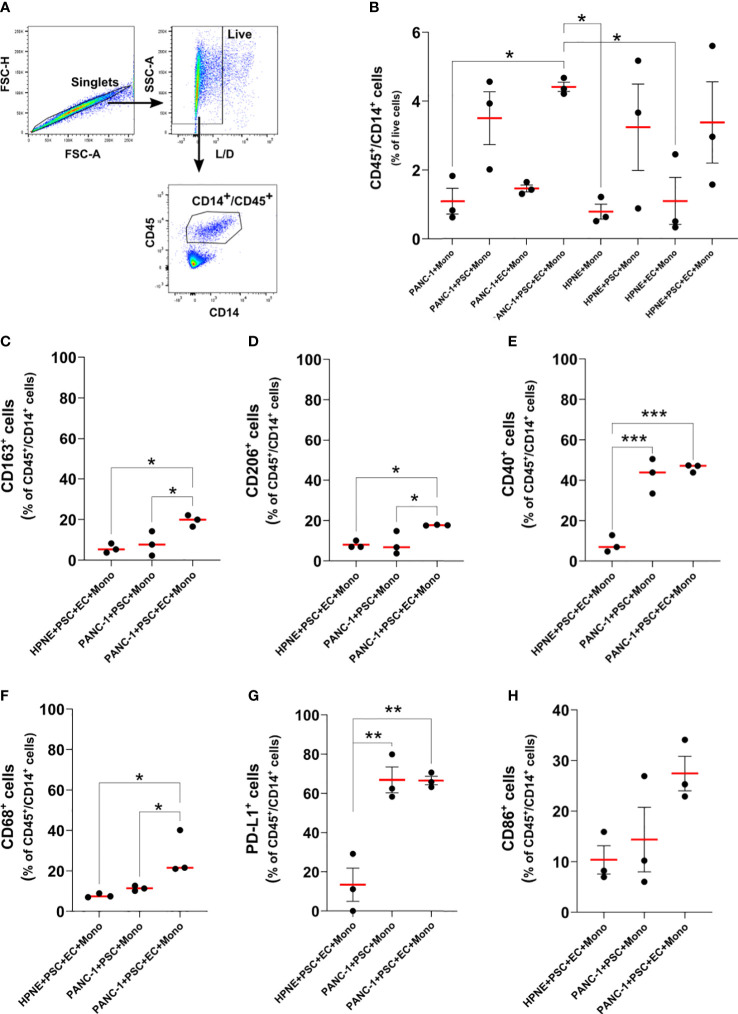
Monocytes differentiate in macrophages with increased expression of M2-like markers, CD40, and PD-L1 in PANC-1 4-culture spheroids. **(A)** Gating strategy of flow cytometry data to identify monocytes as CD45+/CD14+ live single cells. **(B)** Dot plot of the flow data showing the number of CD45+/CD14+ monocyte as percentage of live cells for each PANC-1 and HPNE spheroids. Data are shown as mean ± SEM. Statistical significance for comparisons was determined by one-way ANOVA with Dunnett’s *post-hoc* test. *p <0.05. **(C–H)**. Expression of key macrophage markers for PANC-1 and HPNE 4-culture (PANC-1 4-culture and HPNE 4-culture, respectively) are presented as dot plot of the fold change from the respective FMO intensity (left plot), histograms (center plot) and as dot plot of the percentage of the total number of CD45+/CD14+ monocytes (right plot). Data are shown as mean ± SEM in the dot plots. Statistical significance for comparisons was determined by one-way ANOVA with Dunnett’s *post-hoc* test. *p<0.05, **p<0.01, ***p<0.0001.

Dual ontogeny of macrophages in PDAC TIME has been described by recent studies (16). Both macrophages of fetal and bone marrow origin can be found within the PDAC TIME. To partly reproduce the bone marrow-derived myeloid component of the tumor, we decided to add monocytes to the PANC-1 spheroids. To understand the effect of the TIME on the expression of key markers of differentiation/polarization on monocytes we performed flow-cytometry on cells dissociated from the PANC-1 and HPNE spheroids at day 7 (**Figure 7**). The flow cytometry analysis confirmed our previous observations on PSC supporting role for monocytes survival and embedding within the tumor spheroids (**Figure 4**). The percentage of CD45^+^/CD14^+^ events among the live cells, in fact, increased when monocytes were in co-culture with PSC compared to the monoculture condition, either in PANC-1 or HPNE spheroids. However, the increment of CD45^+^/CD14^+^ monocytes when in co-culture with PSC was statistically significant in PANC-1 spheroids but not in HPNE spheroids.

At day 7 monocyte-derived cells (CD45/CD14^+^ cells) obtained from the 4-culture PANC-1 spheroids showed an increased expression of CD163, CD206, CD40, CD68, and PD-L1 when compared with the same cells obtained from the dissociation of 4-culture HPNE spheroids (**Supplementary Figure 6**). The percentage of monocytes/macrophages expressing CD68, CD163, CD206, PD-L1, CD40 in PANC-1 4-culture spheroids was significantly greater than the one observed in the PANC-1+PSC+Mono spheroids (3-culture) and the HPNE 4-culture spheroids (**Figures 7C–G**). The higher expression of CD68, CD163, CD206 on CD45/CD14^+^ cells in the 4-culture PANC-1 spheroids suggest a monocytes differentiation into M2-like macrophages, known to have tumor supporting functions. The increased expression of PD-L1 on CD45/CD14^+^ cells in the 4-culture PANC-1 spheroids also suggests that these cells may have an immunosuppressive role. The higher expression of the co-stimulatory molecule and M1 marker CD40 on CD45/CD14^+^ cells in the 4-culture PANC-1 spheroids could explain the therapeutic effect of CD40 ligand/agonists in the treatment of PDAC, boosting an anti-tumor response (29, 30).

The percentage of monocyte-derived cells expressing PD-L1 or CD40 was greater in both 4-culture and 3-culture PANC-1 spheroids compared to HPNE 4-culture spheroids. By contrast, the CD45^+^/CD14^+^ cells obtained from the HPNE and PANC-1 4-culture spheroids did not show a statistically significant increase in the expression of the CD86 co-stimulatory molecule, although the average CD86 expression was higher for the PANC-1 spheroids.

Although not statistically significant, also the monocyte-derived cells of the 3-culture PANC-1 spheroids containing PANC-1+PSC+Mono showed an increased expression of CD163 and CD68 when compared with the 4-culture HPNE spheroids. However, in the PANC-1 3-culture spheroids, the percentage of CD68^+^ or CD163^+^ cells in the CD45^+^/CD14^+^ population was not different from the one observed the HPNE 4-culture spheroids.

### PANC-1 4-culture spheroid cytokine signature mimics PDAC patient features

The cytokines released by each cell type present in the TIME affects the proximal (within the TIME) and the distal (plasma) concentrations of molecular signals that participate in the recruitment and activation of cells (31). We, therefore, conceived that a relevant spheroid model will be able to recapitulate the production of key cytokines observed in patients. Current monoculture tumor spheroid models lack the presence of specific cytokines present in the PDAC TIME, such as CCL4/MIP-1β, CCL5/RANTES, CXCL9, and CXCL10 accidentally simulating an immune silent or excluded PDAC TIME. Indeed, these monoculture tumor models are missing the key cellular and humoral interplays responsible for the modulation of cytokine signaling within the PDAC TIME, ultimately leading to immunosuppression or immunoregulation in the context of chronic inflammation (32).

Essentially, shaping PDAC TIME requires various cytokines produced by different cell types from the TIME cooperating with a mutated Ras signaling in pancreatic epithelial ductal cells (31). Established the key role of cytokines in the PDAC TIME, we assessed the levels of some principal cytokines in the supernatant of PANC-1 and HPNE spheroids with increasing cellular heterogeneity to characterize the cytokine expression in our model (**Supplementary Figure 7**) and compare it to PDAC patient samples. In some cases, the cytokine analysis allowed us to identify the type of cell in the spheroid responsible for the changes in the cytokine signaling and potential cellular synergisms that lead to cytokine modulation.

#### M1 macrophage-associated cytokines are less expressed in PANC-1 spheroids compared to HPNE spheroids

The enrichment of M2-like macrophages in the tumor of PDAC patients is usually described as a negative prognostic marker (33). The presence of M2-like macrophages within the TIME inhibits cytotoxic CD8 T-cell functions *via* the reduction of anti-inflammatory cytokines that promote T-cell proliferation and anti-tumor responses (33). M1-like macrophages, on the contrary, have been shown to promote anti-tumor responses mainly *via* the release of pro-inflammatory cytokines which are significantly reduced in M2-like macrophages cultures (34).

**Figure 8:**
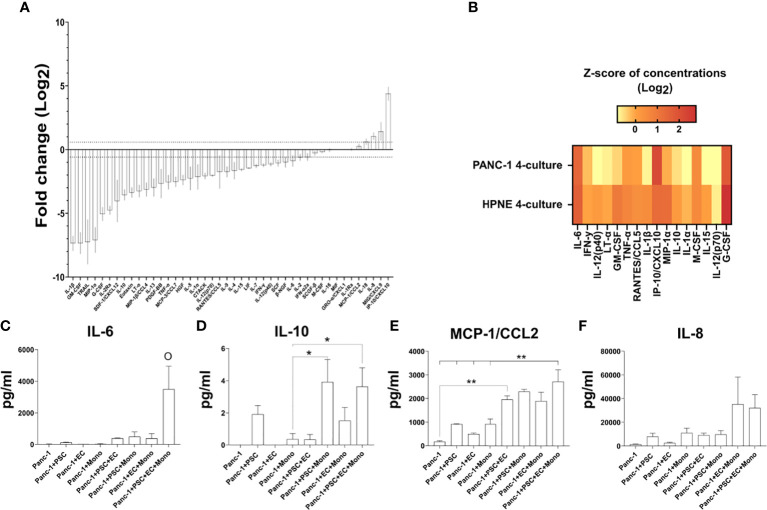
Expression of cytokines in the supernatant of PANC-1 spheroids and their comparison to non-cancerous HPNE spheroids. **(A)** Log2 fold changes of the cytokine concentrations observed in PANC-1 4-culture spheroids at day 7 using the HPNE 4-culture spheroids as control. Dotted lines indicate ± 1.5 fold change. **(B)** Heatmap the multiplex bead-based cytokine analysis of the supernatant of the spheroids at 7 days of culture presenting typical M1-like macrophage-associated cytokines as z-scores of concentrations (Log2). **(C–F)** Bar plots of the concentrations of IL-6 **(C)**, IL-10 **(D)**, MCP-1/CCL2 [**(E)** IL-8 **(F)**] cytokines that have been detected in high concentration in the plasma of patients with poor survival.

Our multiplex bead-based cytokine analysis showed that at day 7 pro-inflammatory cytokines were overall significantly decreased in the supernatant of PANC-1 4-culture spheroids when compared to those of HPNE 4-culture except CXCL10 as shown in **Figures 8A, B**. Among the selected cytokines the greatest and significant decreases between PANC-1 4-culture spheroids and HPNE 4-culture spheroids were observed mainly in the cytokine involved in M1 polarization (**Figure 8B**). In addition to pro-inflammatory cytokines, many of the other cytokines in the panel were significantly downregulated in cancer spheroids compared to HPNE spheroids.

#### PANC-1 4-culture spheroids showed greater concentrations of plasma-detectable cytokines associated with poor prognosis in patients

Among the different cytokines that can be detected in plasma of patients, an increase of IL-6, IL-8, IL-10, CCL2/MCP-1, IP-10/CXCL10 at diagnosis have been associated with poor prognosis of PDAC patients (35–37).

**Figure 9:**
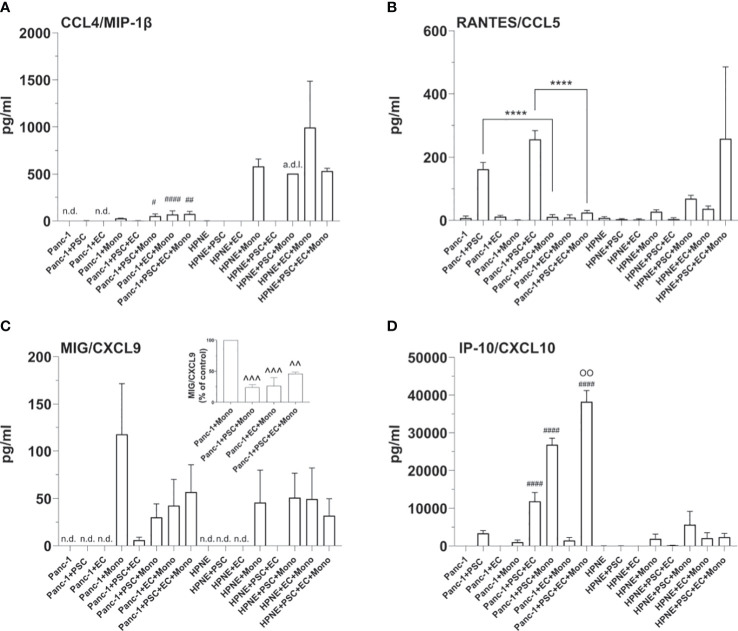
Cellular interplays within the spheroids modulate the expression of T cell recruiting cytokines. Bar plots of the concentrations of T-cell attracting cytokines, i.e. MIP-1β **(A)**, RANTES **(B)**, MIG **(C)**, IP-10 **(D)**. Statistical significance for comparisons was determined by one-way ANOVA with Dunnett’s *post-hoc* test. OO p <0.01 when compared with any of the other tested conditions; ****p<0.0001; ^#^p<0.05, ^##^p<0.01, ^####^p<0.0001 when compared to the HPNE analogue spheroid containing the same type of additional cells; ^^p<0.01, ^^^p<0.001 when compared to PANC-1+Mono spheroids; n.d., non detectable, a.d.l., above detection limit.

Our multiplex bead-based cytokine analysis on the supernatant of *PANC-1 spheroids* at day 7, showed that IL-6, IL-10, IP-10/CXCL10 cytokines were non-detectable (n.d. in **Figures 8C, D**, **9D**), whereas the MCP-1/CCL2 and IL-8 presented very low concentrations (**Figures 8E, F**) in PANC-1 monoculture spheroids. The addition of PSC to the PANC-1 cells with or without EC made IL-6 and IL-10 detectable and increased the concentration of MCP-1/CCL2 (**Figures 8C–E**). The co-culture of monocytes with PSC, EC and PANC-1 cancer cells (PANC-1 4-culture) significantly increased the concentration of IL-6 when compared to any of the spheroids with a lower cellular heterogeneity (**Figure 8C**). By contrast, the PANC-1 4-culture spheroids did not show a significant increase in the concentration of IL-10 and MCP-1/CCL-2 when compared with any other PANC-1 3-culture condition. However, at day 7 the concentrations of MCP-1/CCL2 (**Figure 8E**) in the supernatant of PANC-1 3- and 4-cultures spheroids were significantly higher than the one detected in the samples obtained from PANC-1 2-culture and monoculture spheroids.

Notably, concentrations of the regulatory T cell-recruiting chemokine CXCL10 were significantly increased in PANC-1 4-culture spheroids when compared to any other tested PANC-1 and HPNE condition (**Figure 9D**).

#### 4-culture spheroids chemotactic signals for T cell recruitment are modulated by PANC-1 cells and monocytes

The TIME of PDAC patients with a shorter overall survival is usually classified as “cold”. With this term the scientific community define a tumor lacking T cells infiltration as a result of a low mutation burden, low major histocompatibility complex I (MHCI) expression, and high expression of molecules with immunoregulating or immunosuppressive functions. Immune profiling on PDAC patient samples revealed that accumulation of CD3+ T cells in the tumor correlates with better survival (38). A recent study showed that the recruitment of T cells within the PDAC TIME and the associated anti-tumor response is dependent on the greater co-expression of 4 chemokines: CCL4/MIP-1β, CCL5/RANTES, CXCL9 and CXCL10 (39). In order to study cell-cell interactions within the TIME and to test immunotherapies, a relevant cancer model should simulate the secretion of chemokines involved in the infiltration of T cells (cytotoxic, helper or regulatory) within the tumor as well as mimics modulation of chemotactic and activating factors by myeloid cells, well-known to suppress cytotoxic T cell response in cancer (40).

Concentration of T cell recruiting cytokines were undetectable or very low in both PANC-1 and HPNE monoculture spheroids (**Figures 9A–D**). The addition of monocytes to both PANC-1 and HPNE spheroids resulted in an increase of CCL4/MIP-1β concentrations, significantly lower in PANC-1 spheroids when compared to HPNE analogues (**Figure 9A**). CXCL9 was also detectable in presence of monocytes for both HPNE and PANC-1 spheroids. The comparison of MIG/CXCL9 concentrations between HPNE+Mono and PANC-1+Mono showed that the expression of this cytokine is significantly more concentrated in presence of PANC-1 cells (**Figure 9C**). The presence of either PSC or EC to the PANC-1 spheroids containing also monocytes lowered the concentrations of MIG/CXCL9 up to 23.9% and 26.3%, respectively (**Figure 9C**). The PANC-1 4-culture spheroids, containing PSC, EC, and monocytes did not further decreased the expression of CXCL9 when compared with the PANC-1 3-culture spheroids containing monocytes. The concentrations of RANTES/CCL5 were significantly increased in PANC-1 spheroids containing also PSC when compared to PANC-1 monoculture spheroids. The co-culture of PSC and EC with PANC-1 cells significantly increased the concentrations of RANTES/CCL5 when compared to PANC-1+PSC spheroids (**Figure 9B**). By contrast, the PANC-1 4-culture spheroids containing monocytes showed a significant decrease of RANTES/CCL5 when compared to the same spheroids not containing monocytes, showing concentrations comparable to those detected in PANC-1 monoculture spheroids (**Figure 9B**). The HPNE 4-culture spheroids showed a non-statistically significant increase in the concentration of RANTES/CCL5 when compared with the same spheroids not containing monocytes. The concentrations of the chemokine IP-10/CXCL10 were increased in PANC-1+PSC spheroids and further increased with higher cellular heterogeneity reaching the greater concentration in PANC-1 4-culture spheroids (**Figure 9D**). PANC-1+EC concentration of IP-10/CXCL10 were not detectable as observed in PANC-1 monoculture spheroids whereas PANC-1+Mono spheroids had 1/3 of the concentration of IP-10/CXCL10 when compared with PANC-1+PSC. Within the PANC-1 spheroids, the combination of PSC with monocytes or EC clearly showed a synergism of IP-10/CXCL10 secretion which is not the mere sum of the concentrations observed in each 2-culture condition (PANC-1+PSC, PANC-1+EC, PANC-1+Mono) (**Figure 9D**). In HPNE spheroids, the detection of IP-10/CXCL10 was only associated to the presence of monocytes and was not detectable in HPNE+PSC. Overall, the analysis of the cytokine/chemokine expression shows how the PANC-1 4 culture spheroids intrinsically contains the potential of expressing crucial chemokines contained in the PDAC TIME and the interplay among the different cell types recreates an immunoregulation on those expressed cytokines.

## Discussion

To increase the number of effective therapies for the treatment of PDAC reaching clinical trials, recapitulating the TIME in preclinical models is imperative. Tumor preclinical models should be designed to reproduce the composition of PDAC TIME as closely as possible, posing particular attention to mimicking the intra- and inter-cellular interplays occurring in PDAC TIME of patients who do not respond to existing therapies. Currently, the available preclinical models either do not consider interspecies differences (animal models) or do not reasonably reproduce the TIME of PDAC, lacking specific cell types and their 3D spatial organization. The PDAC TIME is densely populated with non-cancer cells like endothelial, stellate, and immune cells, which have been shown to interact with each other shaping the TIME and playing a critical role in establishing therapy resistance (16, 41, 42). Stem cell-derived organoids serve as essential *in vitro* models for investigating cancer cell mechanisms, especially because they recapitulate cell lineages often organized in functional units like organs. Regrettably, despite extensive efforts to establish heterocellular organoids, further work is necessary to comprehensively represent the cellular interactions among stromal, immune and endothelial cells within the TIME (43). Recent studies characterized 3D hetero-cellular tumor spheroids models, accounting for different PDAC stroma cell types. However, none simultaneously co-cultured the four most abundant and critical cells in PDAC TIME: cancer cells, endothelial cells, pancreatic stellate cells, and macrophages. In recent years our work focused on improving 3D *in vitro* models by incorporating complexity that is fundamental in understanding the tumor microenvironment and the immune system interaction (44–48).

In this study, we formed and characterized, for the first time, viable PANC-1 tumor spheroids containing human endothelial cells, pancreatic stellate cells, and blood-derived monocytes. The non-cancerous cell types (PSC, EC, and monocytes) were well represented on day 7, especially in the 4-culture tumor spheroids. The percentage of EC was low, although these cells demonstrated the capability to interact with each other, forming 3D endothelial networks similar to the compressed vessels observed in murine models of PDAC and histological analysis of the patient tumors (49, 50). The difference observed in the 3D endothelial network arrangement of PANC-1 and HPNE spheroids suggests that the co-culturing of EC and cancer cells significantly impacts the formation of the EC structures. Most importantly, the EC network was only observed in tumor spheroids containing the combination of PSC and EC, suggesting a critical pro-angiogenic interplay between these two cell types, but also an anti-angiogenic effect exerted by the cancer cells as previously demonstrated by Di Maggio et al. (50). Although it cannot be entirely ruled out that the disrupted EC structures observed in PANC-1 spheroids are partly attributable to EC death, it is worth considering that the typical features of the TIME, such as lack of substrates and the elevated production of reactive oxygen species, might have additionally contributed to the reduced proliferation of these cells, as previously demonstrated (51–53). The volume of the 3D endothelial network observed in our tumor 4-culture spheroids was also significantly reduced compared to the same tumor spheroids lacking monocytes. We can speculate that the increased collagen I deposition observed in the tumor 4-culture spheroids physically compresses the EC network, as demonstrated by different studies performed in murine models of PDAC (27, 54, 55). In these studies, the decrease in collagen I deposition or inhibition of enzymes deputed to collagen crosslinking was associated with the decompression of the vessels. On the other hand, other physical and molecular factors due to the presence of monocytes in the tumor 4-culture spheroids may have contributed to the decreased volume of the EC network. Additional experiments reducing the deposition of collagen I or its crosslinking may clarify the cause of the reduced EC network volume in tumor 4-culture spheroids.

It has been shown that vascular structures develop in an environment in which perivascular cells, such as PSC, are present (50, 56). Therefore, we assessed the distance between EC and PSC in our tumor spheroids. Through our spatial analysis, we confirmed that PSC surrounded EC and that monocyte-derived macrophages were also localized in the proximity of the endothelial structures. Of note, macrophages have been localized in the stroma of PDAC, where PSC and EC were located in orthotopic murine tumor models (16), similar to what was observed in our tumor spheroids. The spatial localization of these three cell types (PSC, EC, and monocytes) in the core of the tumor spheroids seems to rely mainly on the presence of the PSC that localize in the core of the tumor spheroids even when co-cultured alone with cancer cells. In human and murine PDAC, activated PSC are the main cells producing the stiff fibrotic ECM leading to desmoplasia and hypoxia. Hypoxia is usually observed in the core of spheroids larger than 200 µm (57), mainly through the nuclear translocation of HIF-1α. It promotes the secretion of several cytokines involved in the recruitment of fibroblasts and immune cells (58), potentially explaining the localization of PSC and monocytes within the core of our tumor spheroids.

When we assessed the expression and nuclear translocation of the transcription factor HIF-1α as a key regulator of the hypoxic response, we noticed HIF-1α expressed with two different intensities. The high-intensity HIF-1α was mainly located in the nuclei of cells in the core of the spheroids. From the flow cytometry analysis, it was possible to discriminate that these cells with a higher intensity were both monocytes and PANC-1 cells (**Figure 5E**). To rule out the possibility that the size of the spheroids rather than the cell composition was affecting the HIF-1α expression, we compared the monoculture and 4-culture tumor spheroids having about the same size. Notably, the HIF-1α expression was significantly lower in monoculture spheroids when compared with 4-culture spheroids underlining that the cell composition was critical for the increased HIF-1α expression and nuclear translocation.

Another effect of the PSC presence in the tumor spheroids was an increased size and number of cancer cells counted in our tumor spheroids, probably associated with the higher proliferation observed on day 4 and in agreement with other *in vitro* studies (15, 59). Instead, for the heterocellular tumor spheroids without PSC, the increased number of cancer cells could be attributed to an improved embedding of the cancer cells within the spheroids.

Although PSC-induced proliferation represents an interplay between cancer cells and stellate cells, other fibroblast populations in the PDAC TIME may contribute to cancer cell proliferation, as observed in a recent study of PSC depletion in an orthotropic mouse model (10). By contrast, the resistance to radiotherapy in PDAC patients (60) seems specifically induced by PSC (15). Mantoni et al. demonstrated that only human PSC and not MRC-5 confer radiotherapy resistance to pancreatic cancer cells, reinforcing the importance of developing a PDAC TIME model containing stellate cells of pancreatic origin.

In addition to affecting cancer cell functions, PSC depletion decreased the number of macrophages within the tumor (10). Accordingly, we observed that the monocyte percentage in PANC-1 spheroids increases with PSC, suggesting that PSC-monocyte interplay observed in *in vivo* models may occur in our *in vitro* model. Characterizing the monocytes embedded within the spheroids with different compositions further demonstrated that interaction with each cell type contributed to the expression of key macrophage polarization markers and the immune checkpoint PD-L1. We observed how the polarization of monocytes toward an M2-like macrophage phenotype occurs only in the PANC-1 4-culture at 7 days after seeding, as demonstrated by the increased expression of CD68, CD163, and CD206 markers in the spheroids with higher cellular heterogeneity. In line with this observation, the PANC-1 4-culture spheroids better mimic the M2 macrophage-populated PDAC TIME of patients with poorer prognoses (61). Differently, the expression of critical immune regulators, namely PD-L1 and the M1 marker CD40, on monocytes/macrophages in our tumor spheroids depended on their co-culture with PANC-1 cells and PSC. At the same time, we did not observe any significant increase in the expression of either CD40 or PD-L1 in monocytes/macrophages within HPNE spheroids. Similarly, Kung et al. elegantly showed in their *in vitro* experiments (62) that under the influence of PSC, cancer cells increased the secretion of S100A9, causing the increased expression of PD-L1 on monocytes/macrophages.

The increased percentage of M2-like macrophages in our 4-culture tumor model well reflects the decreased concentrations of typical M1 cytokines detected in the PANC-1 spheroids compared to the HPNE spheroids (**Figure 8**). The supernatant of HPNE spheroids showed high concentrations of several pro-inflammatory cytokines, most probably due to VEGF stimulation and cellular stress occurring during the formation of the spheroids. It has been demonstrated that VEGF promotes the release of inflammatory cytokines (such as IL-6, IL-8/CXCL-8, and GRO- α/CXCL-1) by endothelial cells through VEGF receptor 2 activation. In turn, inflammatory cytokines such as TNF-α, IL-1β, IL-6, and IL-8/CXCL-8 induce VEGF expression, reinforcing angiogenesis and inflammation (63). The HPNE spheroids served as a control of inflammation to understand how the key cellular players in the TIME modulate the secretion of key inflammatory cytokines. In the HPNE spheroids, we observed multiple cell interplays leading to positive and negative regulation of cytokines. It was clear that pro-inflammatory cytokines were concentrated in HPNE spheroids, but cancer cells contributed to reducing the concentration of typical Th1 cytokines (e.g., TNF, IFN-y, IL-12). From the cytokine analysis, we concluded that multicellular interactions within the tumor spheroids determine significant changes in the cytokine pattern, leading to the polarization of monocytes into an M2-like phenotype. The tumor spheroids with the higher degree of cellular heterogeneity (4-culture) better resemble the cytokine signature observed in the plasma of patients with poor survival and not responding to therapies (36, 37), making this model suitable for further investigations to identify new strategies for reprogramming the PDAC TIME. Strikingly, CCL2/MCP-1, well known to mediate the recruitment of monocytes in the hypoxic areas (64), were more concentrated in 3- and 4-culture tumor spheroids, supporting our results on the spatial localization of monocytes in the hypoxic area of the spheroid.

We also assessed the expression of key cytokines involved in T-cell recruitment and activation in PDAC patients (39). These cytokines were highly affected by the presence of the different cell types. Briefly, we could observe that the concentrations of these cytokines (mainly MIP-1β/CCL4, RANTES/CCL5, MIG/CXCL9) were kept low in PANC-1 4-culture spheroids as a result of the combined cell types. In the tumor spheroids, we could establish the role of each cell type on cytokine secretion by assessing their concentrations in spheroids missing one of the cell types. T cell recruiting cytokines identified by Romero and collaborators were mainly suppressed by monocytes and PSC within the aggregate, reinforcing the importance of cellular heterogeneity in spheroid models of PDAC.

Interestingly, IP-10/CXCL10 was the only T-cell-related cytokine among the 4 suggested by Romero et al., which was increased in the PANC-1 spheroids. Notably, in more than one study, high plasma concentrations of IP-10/CXCL10 have been associated with decreased survival in PDAC patients (19, 37). Lunardi et al. showed that IP-10/CXCL10 was increased in the co-culture of the PSC with pancreatic cancer cells. IP-10/CXCL10 has been shown to be a chemoattractant for T cells, including CD4-Th1, CD8, and Tregs (65, 66). In particular, the increased concentrations of IP-10/CXCL10 in the TIME of pancreatic cancer have been linked to the recruitment of Tregs to the tumor site (19), which, in turn, may contribute to disease progression (67). This increase was also observed in our experiments and IP-10/CXCL10 synergistically increased in the presence of EC and/or monocytes. Lunardi et al. also demonstrated that the patients’ higher expression of IP-10/CXCL10 was associated with a denser stroma. Accordingly, with these clinical data, in our PANC-1 4-culture spheroids, we observed an increased expression of collagen I associated with increased secretion of IP-10/CXCL10. Although collagen I is not the only component of the dense PDAC stroma, it has been shown to play a key role in the induction of the desmoplastic response (26). Therefore, the increased collagen I deposition observed in our PANC-1 4-culture spheroids sustains another key similarity between our tumor model and the PDAC TIME of patients.

In conclusion, our PANC-1 4-culture spheroids represent a promising tool for studying cell-cell interactions in the PDAC TIME. Future studies may also address if the PANC-1 4-culture spheroids are recapitulating the increased infiltration of Treg as shown in the *in vitro* model of Lunardi et al. More broadly, PANC-1 spheroids could be helpful in functional assays of T and myeloid cell infiltration (68, 69) to observe if the tumor spheroids induce chemotaxis and immune responses similar to those observed in patients, as confinement, anergy, and immunosuppression. Although this study aims to develop a tool for studying cell interactions in the PDAC TIME, it is important to exercise caution regarding potential therapeutic testing using this system. We foresee further research studies with our PDAC spheroids with different cellular heterogeneity embedded in microfluidic devices to test their sensitivities to established treatments (chemotherapy, radiotherapy, and immunotherapies) (70), aiming to validate the model for drug screening. The PANC-1 4-culture spheroids, as described in this study, being an *in vitro* tumor model, come with intrinsic limitations when compared to a patient’s tumor. Although the increased number of degrees of freedom resulting from the heterogeneity of the spheroids might represent limitations for mechanistic studies using this model, we believe that leveraging advanced spatial transcriptomics and proteomics techniques will enable the extrapolation of crucial molecular changes. This will facilitate the study of mechanisms that were previously difficult to reproduce or assess in other tumor models. Importantly, our model still lacks a poorly perfusable tumor-associated vasculature and mimicking the pathophysiological recruitment of immune cells as observed in *in vivo* models. With an opportune tuning of the culture conditions, patient-derived vascularized heterocellular PDAC spheroids could be developed to answer patient-specific questions bridging preclinical and clinical research.

## Data availability statement

The original contributions presented in the study are included in the article/**Supplementary Material**. Further inquiries can be directed to the corresponding authors.

## Author contributions

Contributions by authors in publication: GG, SIgN, conceived and designed the analysis, collected the data, contributed data or analysis tools, performed the analysis, wrote the paper. GT, SIgN, contributed to perform analysis. AP, IMCB, contributed to designing the analysis. GA, SIgN, initiated, directed and supervised the study, co-designed the experiments and contributed in writing the manuscript. All authors contributed to the article and approved the submitted version.
