# Supplementary material for: Characterization of 3D heterocellular spheroids of pancreatic ductal adenocarcinoma for the study of cell interactions in the tumor immune microenvironment
Source: Front Oncol. 2023 Jul 14;13:1156769. doi: 10.3389/fonc.2023.1156769 (PMC10375712; doi:10.3389/fonc.2023.1156769)
Supplement: Supplementary file 1 [file DataSheet_1.docx]

Supplementary Material

Article Title

**Characterization of 3D heterocellular spheroids of pancreatic ductal adenocarcinoma for the study of cell interactions in the tumor immune microenvironment**

Giulio Giustarini^1*^, Germaine Teng^1^, Andrea Pavesi^2,3^, Giulia Adriani^1,4*^

^1^Singapore Immunology Network (SIgN), Agency for Science, Technology and Research (A*STAR), Singapore, Singapore
^2^ Institute of Molecular and Cell Biology (IMCB), Agency for Science, Technology and Research (A*STAR), Singapore, Singapore
^3^ Mechanobiology Institute, National University of Singapore, Singapore, Singapore

^4^Department of Biomedical Engineering, National University of Singapore, Singapore, Singapore

*** Correspondence:**Giulio Giustarini
[Giulio_giustarini@immunol.a-star.edu.sg](mailto:Giulio_giustarini@immunol.a-star.edu.sg)

Giulia Adriani

[Giulia_adriani@immunol.a-star.edu.sg](mailto:Giulia_adriani@immunol.a-star.edu.sg)

**b**

**a**

**HPaSteC**

**PANC-1**


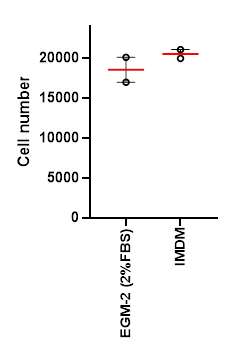

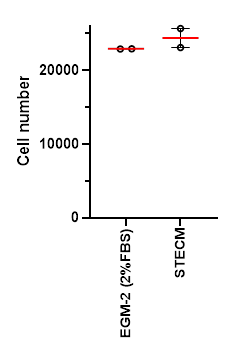


**d**

**c**

**HPNE**

**Monocytes**


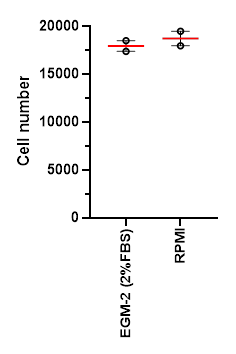

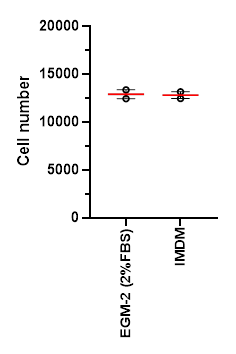


**Supplementary Figure 1. PANC-1, HPaSteC, HPNE and monocyte cell counts after exposure to EGM-2**

Individual value dot plots showing the cell count of (**A**) PANC-1, (**B**) HPaSteC, (**C**) monocytes and (**D**) HPNE after exposure to EGM-2 medium when compared to the recommended media of each cell type. Tests were performed in duplicate.

$$l/2$$

$$l/2$$

$$l$$

**Inner**

**Outer**

**Supplementary Figure 2. Identification of an inner and outer region in spheroids.**

We assessed the spatial cell distribution within the spheroids by dividing the spheroid in two concentric areas: an outer (OUT) and an inner (IN) area. We defined the inner area as the circle having half of the total spheroid radius (l) and having the same centre of the spheroid. The remain area of the spheroid was considered the outer area (Supplementary Figure 1). In these areas we calculated the area under the curve (AUC) of the fluorescent signal of each cell type. The AUC IN/OUT ratio of the fluorescent signal of each cell type provides an understanding of the preferred location of the cells within the spheroid.


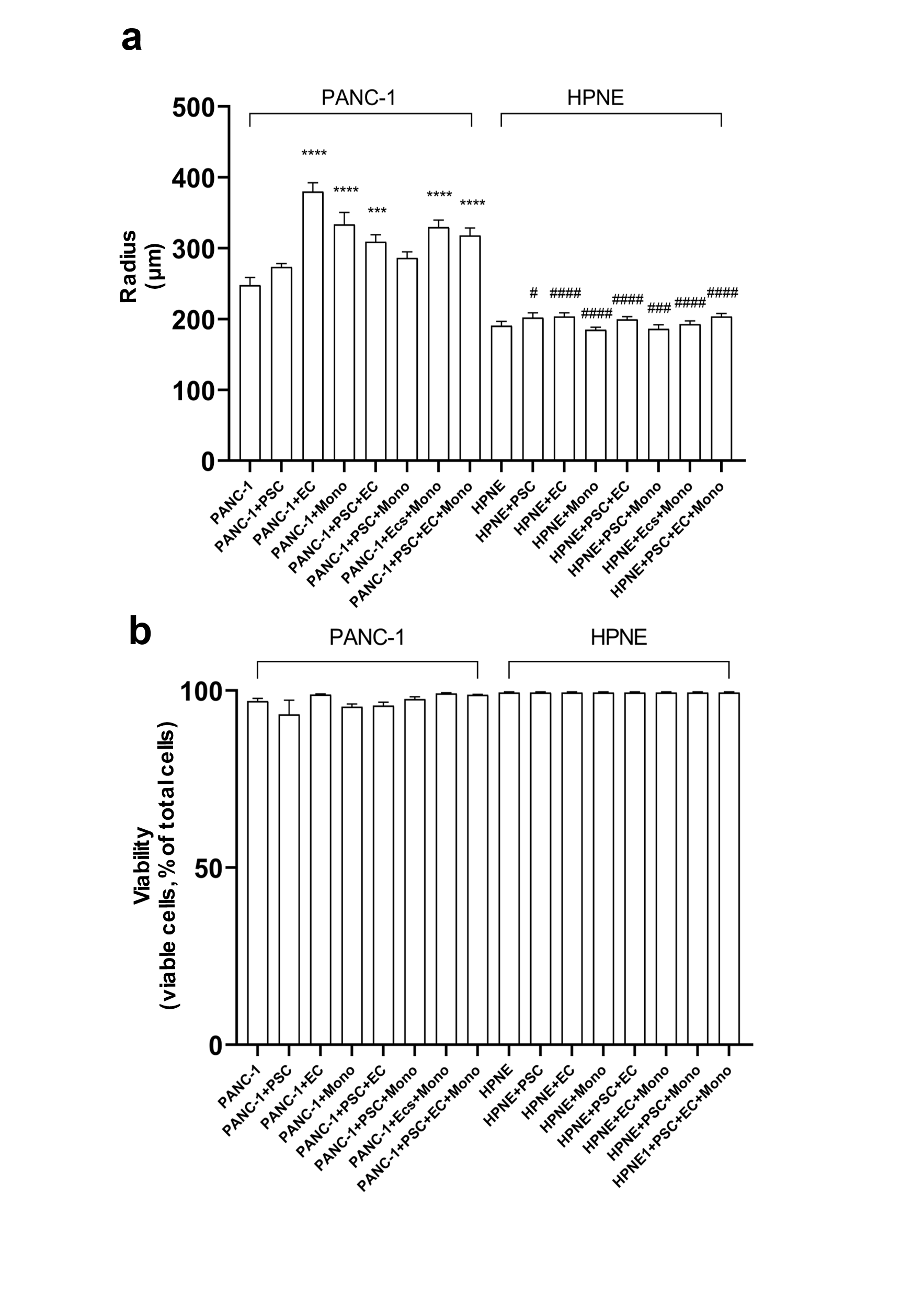
**Supplementary Figure 3. Size and viability of PANC-1 and HPNE spheroids.**

**(A)** Bar plot of the radius of HPNE and PANC-1 spheroids at day 7. Data are shown as mean ± SEM. Statistical significance for comparisons was determined by one-way ANOVA with Dunnett's post-hoc test. **** p < 0.0001 *** p < 0.001 when compared to PANC-1 monoculture spheroids, # p < 0.05, ### p <0.001, #### p <0.0001 when compared with the correspondent PANC-1 spheroid analogue containing the same additional composition of cells. **(B)** Bar plot of the percentage of viable cells at day 7 on the total amount of cells within the spheroid. Viability was determined using DRAQ7 staining to identify cells with an impaired cell membrane integrity. Data are shown as mean ± SEM. Statistical significance for comparisons was determined by one-way ANOVA with Dunnett's post-hoc test.


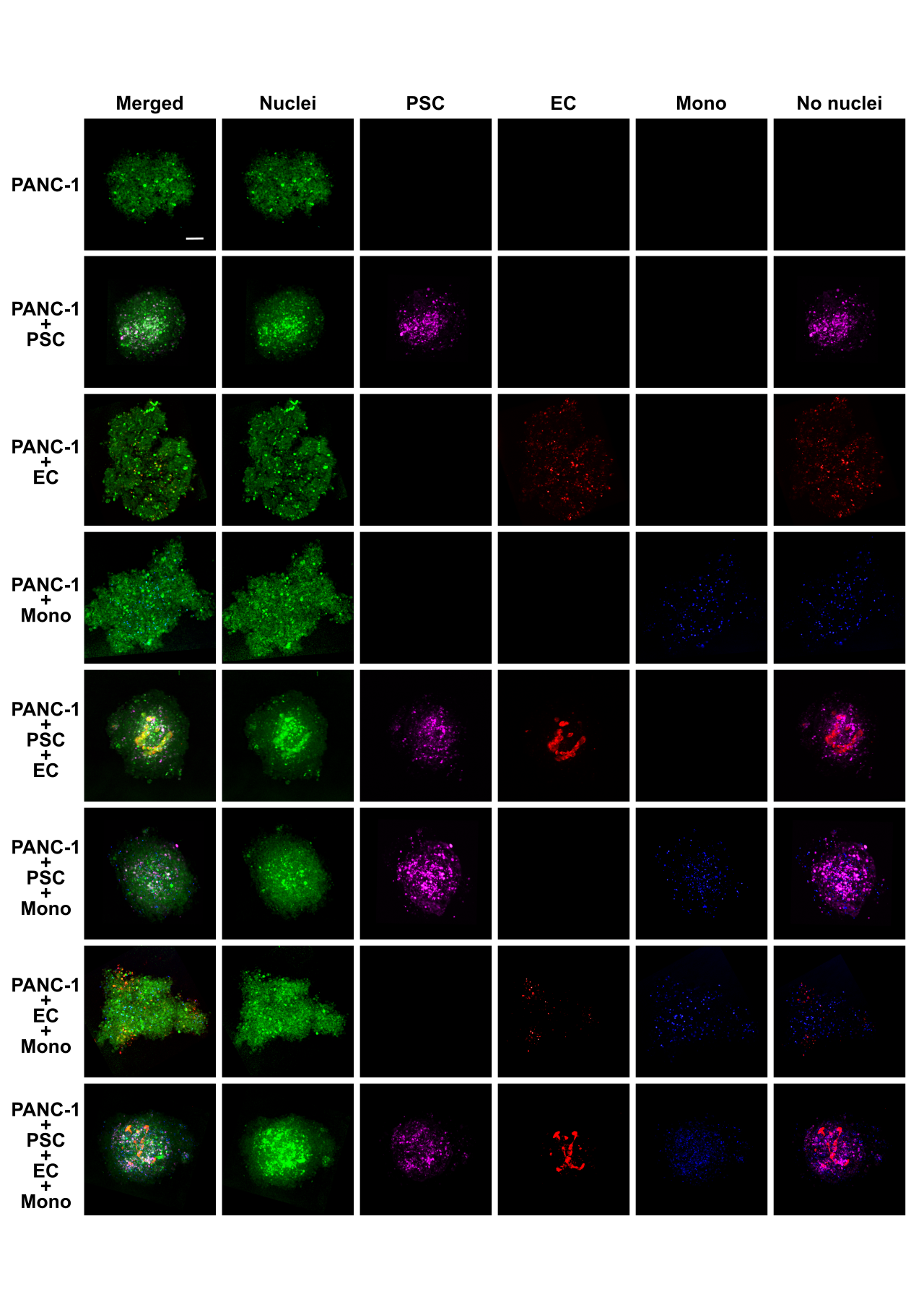


**Supplementary Figure 4. PANC-1 spheroids with different heterogeneity.**

PANC-1 cells were combined with PSC, EC and monocytes following the ratio shown in Table.1 Representative images of PANC-1 spheroids at day 7 using confocal imaging (green: nuclei, purple: PSC, red: EC, blue: monocytes). Scale bar: 100 μm.


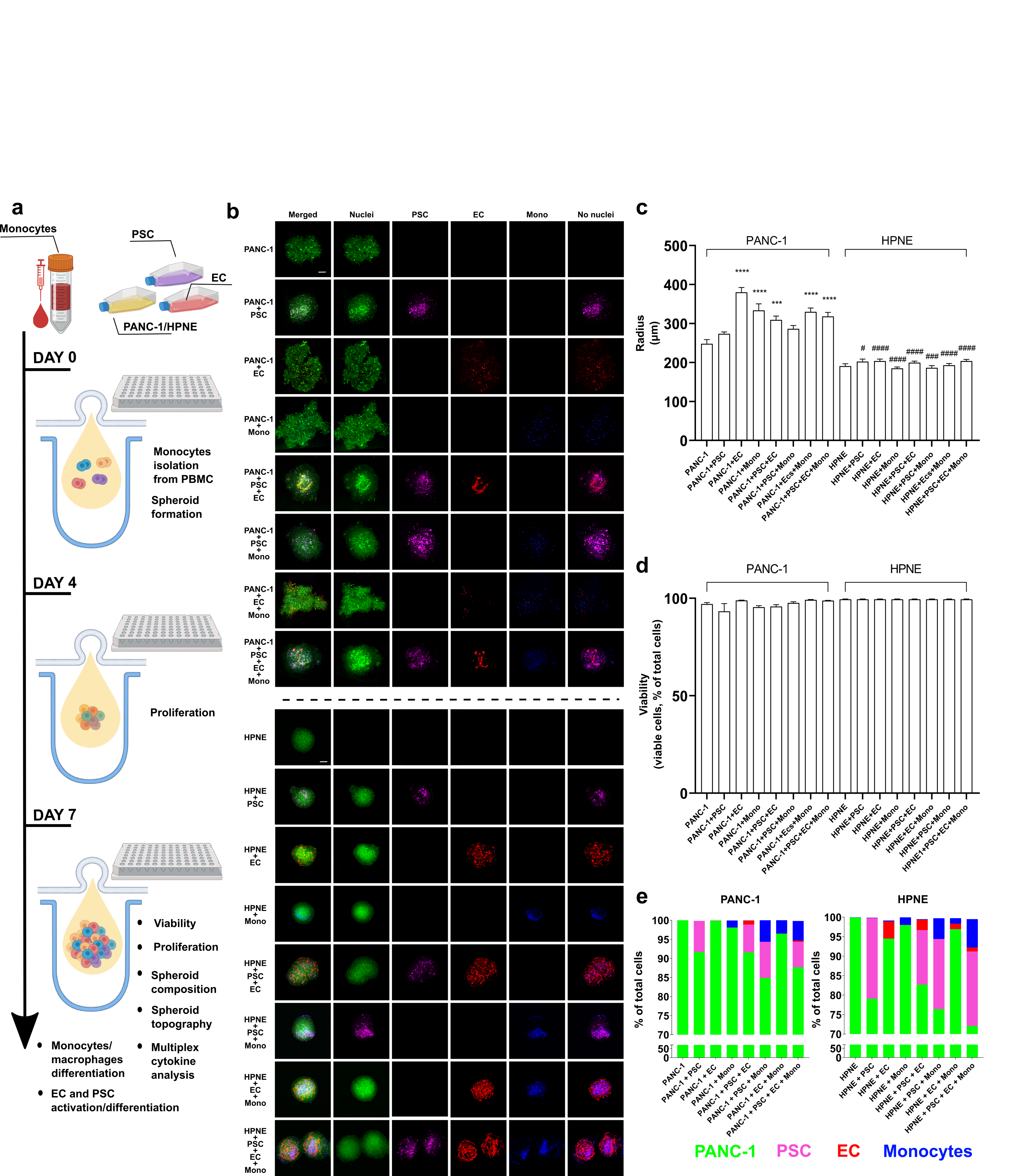


**Supplementary Figure 5. HPNE spheroids with different heterogeneity.**

HPNE cells were combined with PSC, EC and monocytes following the ratio shown in Table.1 Representative images of HPNE spheroids at day 7 using confocal imaging (green: nuclei, purple: PSC, red: EC, blue: monocytes). Scale bar: 100 μm.


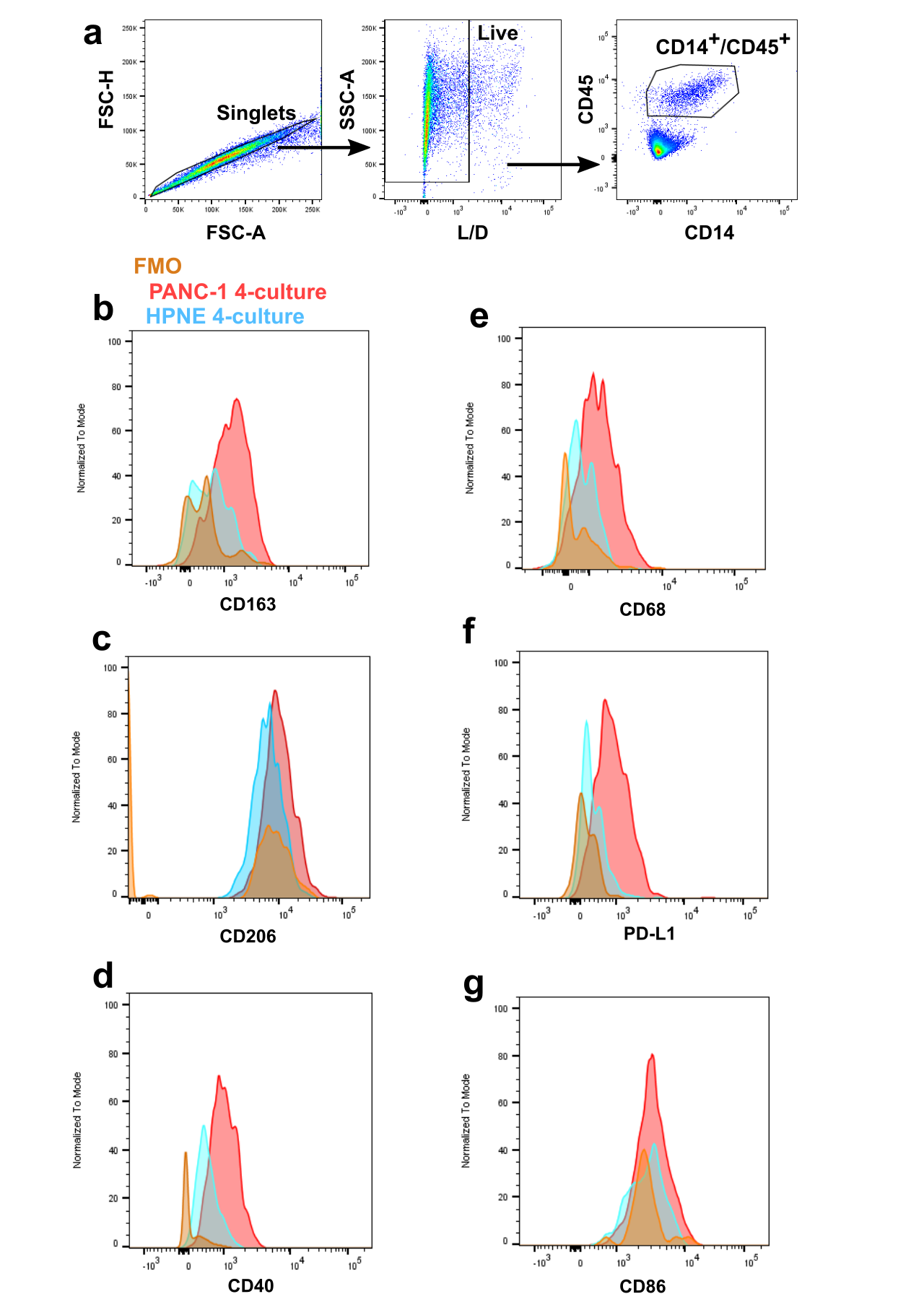


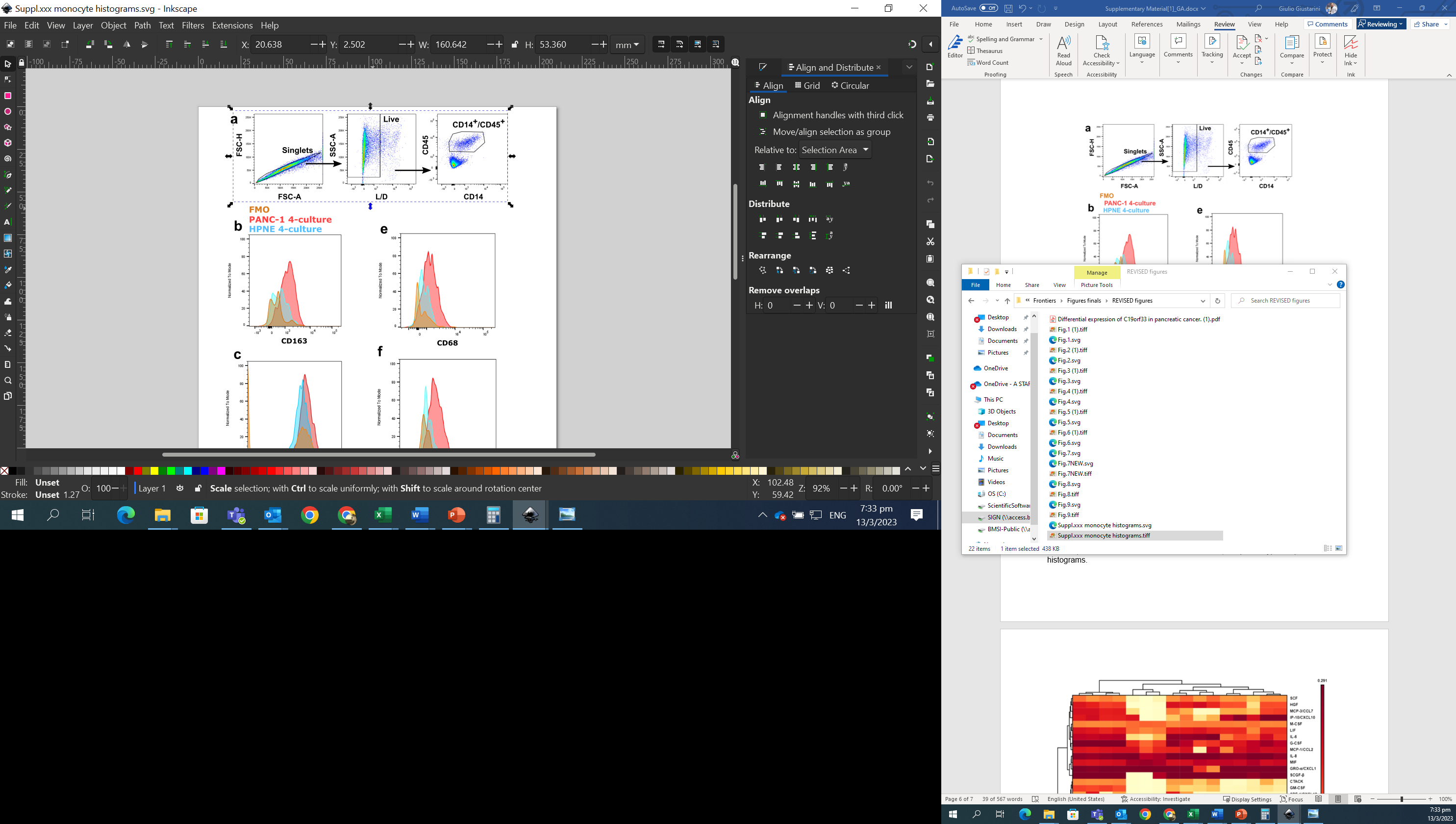


**Supplementary Figure 6. Expression of M1/M2-like markers and PD-L1 in PANC-1 4-culture spheroids.**

**(A)** Gating strategy of flow cytometry data to identify monocytes as CD45+/CD14+ live single cells. **(B)** Expression of key macrophage markers for PANC-1 and HPNE 4-culture (PANC-1 4-culture and HPNE 4-culture, respectively) are presented as histograms.


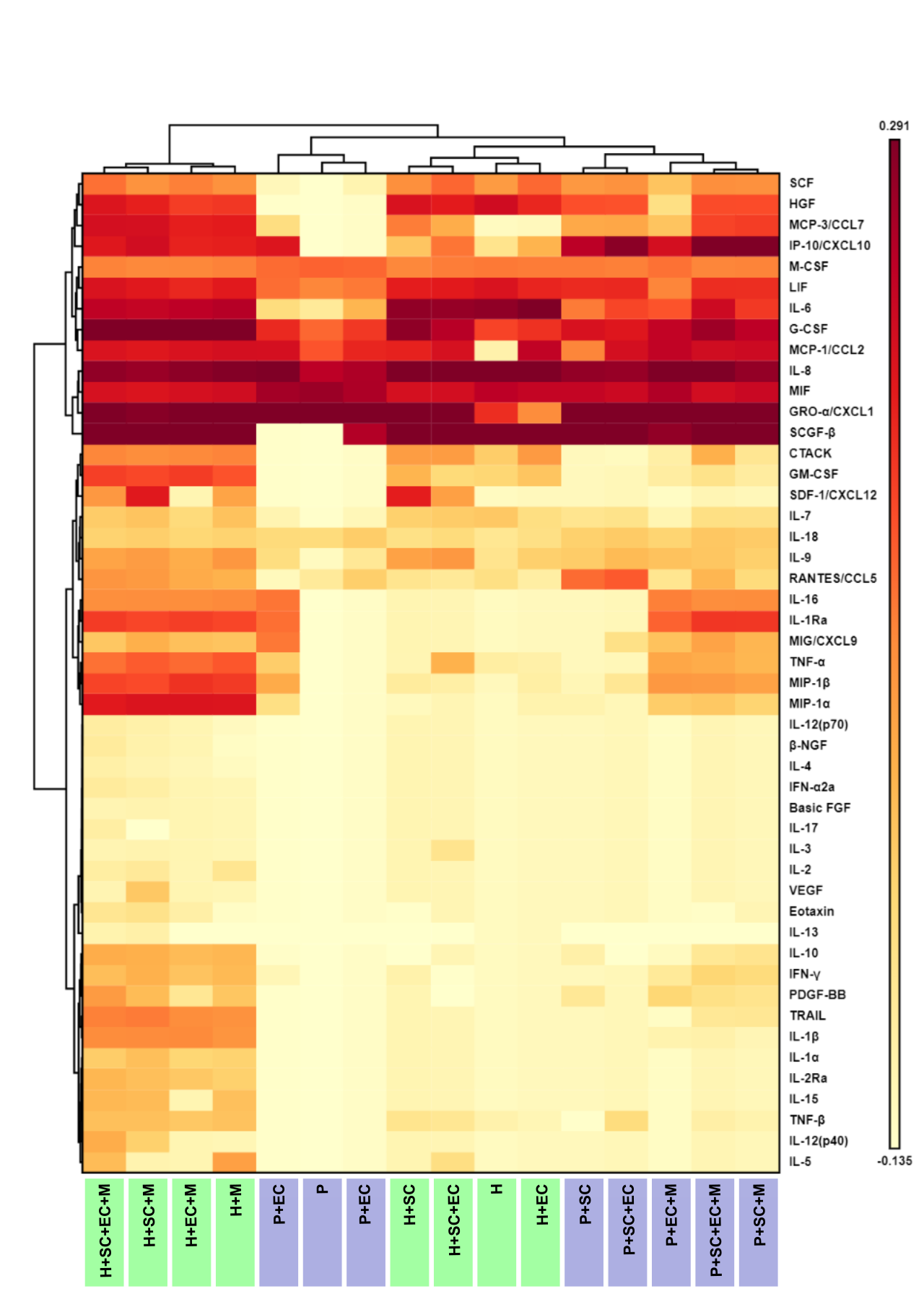


**Color key**

**(Z-scores)**

**Supplementary Figure 7. Hierarchical clustering of spheroids using the expression of cytokines in supernatant at day 7.**

Supernatants of the different spheroids was collected at day 7 and multiplex bead-based assay for the identification of 48 cytokines was performed. Unsupervised hierarchical clustering of the Log2 concentration Z-scores of all the detected cytokine is presented. The values correspondent to the lower limits of detection were used in case of values below the detection limit.
